# Ischemia-induced cleavage of OPA1 at S1 site aggravates mitochondrial fragmentation and reperfusion injury in neurons

**DOI:** 10.1038/s41419-022-04782-0

**Published:** 2022-04-08

**Authors:** Xiang Li, Haiying Li, Zhongmou Xu, Cheng Ma, Tianyi Wang, Wanchun You, Zhengquan Yu, Haitao Shen, Gang Chen

**Affiliations:** 1 Department of Neurosurgery & Brain and Nerve Research Laboratory, The First Affiliated Hospital of Soochow University, 188 Shizi Street, Suzhou, Jiangsu Province 215006 China; 2 Institute of Stroke Research, Soochow University, Suzhou, China

**Keywords:** Stroke, Cell death in the nervous system

## Abstract

Neuronal mitochondrial dynamics are disturbed after ischemic stroke. Optic atrophy 1 (OPA1) and its GTPase activity are involved in maintaining mitochondrial cristae and inner membrane fusion. This study aimed to explore the role of OMA1-mediated OPA1 cleavage (S1-OPA1) in neurons exposed to cerebral ischemia and reperfusion. After oxygen-glucose deprivation (OGD) for 60 min, we found that mitochondrial fragmentation occurred successively in the axon and soma of neurons, accompanied by an increase in S1-OPA1. In addition, S1-OPA1 overexpression significantly aggravated mitochondrial damage in neurons exposed to OGD for 60 min and 24 h after OGD/R, characterized by mitochondrial fragmentation, decreased mitochondrial membrane potential, mitochondrial cristae ultrastructural damage, increased superoxide production, decreased ATP production and increased mitochondrial apoptosis, which was inhibited by the lysine 301 to alanine mutation (K301A). Furthermore, we performed neuron-specific overexpression of S1-OPA1 in the cerebral cortex around ischemia of middle cerebral artery occlusion/reperfusion (MCAO/R) mice. The results further demonstrated in vivo that S1-OPA1 exacerbated neuronal mitochondrial ultrastructural destruction and injury induced by cerebral ischemia-reperfusion, while S1-OPA1-K301 overexpression had no effect. In conclusion, ischemia induced neuronal OMA1-mediated cleavage of OPA1 at the S1 site. S1-OPA1 aggravated neuronal mitochondrial fragmentation and damage in a GTPase-dependent manner, and participated in neuronal ischemia-reperfusion injury.

## Introduction

Approximately eight million people worldwide suffer from ischemic stroke each year, with a mortality rate of about 10%. In the 90% of patients with ischemic stroke who survive, half have neurological impairment and a quarter are severely disabled [1, 2]. The prognosis of ischemic stroke patients is significantly affected by the limited time window for recanalization therapy, as well as by reperfusion injury [3–5].

ATP, produced by mitochondrial aerobic respiration, is the energy source for neurons to maintain cellular homeostasis [6]. Mitochondria are the core organelles that regulate neuronal death, mainly including neuronal apoptosis, necrosis, and ferroptosis [7]. In a rat model of transient occlusion of middle cerebral artery, ATP depletion occurred in the ischemic core and penumbra. After reperfusion, the concentration of ATP in the penumbra of the cerebral cortex briefly recovered, but energy was depleted several hours later, accompanied by neuronal death in the penumbra [8]. Delayed neuronal death after cardiac arrest and resuscitation in humans confirmed the phenomenon of secondary neuronal death after reperfusion [9]. In the above animal and human ischemic stroke events, secondary energy depletion and neuronal death persisted after reperfusion restored oxygen and glucose supply, suggesting mitochondrial dysfunction. Neuronal mitochondrial dysfunction is a key factor that limits the reperfusion time window, leading to reperfusion injury [10].

Neurons are energy-intensive cells with long lives that have sophisticated mitochondrial quality control [11]. Mitochondrial dynamics are important for quality control of neuronal mitochondria [12]. Mitochondrial dynamics include two processes: mitochondrial fission and fusion. Dynamic protein-associated protein 1 (Drp1) plays an important role in neuronal mitochondrial fission, regulated by phosphorylation at sites 616 and 637. Optic atrophy protein 1 (OPA1) is an important executive factor of neuronal mitochondrial fusion. OMA1 and Yme1l mediate the OPA1 splicing to produce S1-OPA1 and S2-OPA1, respectively. Under normal physiological conditions, mitochondrial fission and fusion maintain the normal mitochondrial function by promoting clearance of damaged mitochondria and ATP synthesis, respectively [11]. Abnormal dynamic balance of mitochondria leads to mitochondrial over-fusion or over-division, which will lead to abnormal mitochondrial energy metabolism, increased membrane permeability, release of cytochrome C, and eventually cell death [13, 14]. Lethal excessive fission of the mitochondria occurs in neurons after OGD/R or ischemia-reperfusion [15–17]. However, the regulatory mechanism of neuronal mitochondrial dynamics in cerebral ischemia-reperfusion injury is still not fully understood.

Kumar et al. found that mouse hippocampal neuron cell line, HT22, undergoes lethal mitochondrial fission during OGD/R [15]. The first rapid fission occurs in the early stage of OGD (lasting 20 min), and reoxygenation triggers rapid fusion (lasting 20 min). This is followed by a reoccurrence of excessive mitochondrial fission and failure to fusion, accompanied by destruction of the mitochondrial cristae structure, cytochrome C release, and cell death. This study suggested that rapid mitochondrial fission induced by transient ischemia occurred in the early stage of reperfusion. Excessive mitochondrial fission after reperfusion leads to the destruction of the mitochondrial structure, which is lethal and irreversible. Existing studies reported that the inhibition of Drp1 by gene intervention or inhibitor preconditioning effectively reduces the excessive fission of neuronal mitochondria and neuronal damage caused by cerebral ischemia reperfusion [18–20]. Oxidative stress and excitatory toxicity are two important factors leading to neuronal reperfusion injury [21]. Stefan reported that mitochondrial fission sensitizes neurons to oxidative stress and excitotoxicity [22]. Sundararajan proposed that ischemia initiates mitochondrial damage, making it difficult for the mitochondria to resist reperfusion, and may even aggravate reperfusion injury [14]. In conclusion, changes in neuronal mitochondrial dynamics induced by ischemia may be key factors that limit the recanalization time window and exacerbate reperfusion injury. Therefore, the mechanism of these changes merits further attention.

Recent studies have shown that stress-induced OPA1 processing by OMA1 converts OPA1 into S1-OPA1, which inhibits fusion and triggers mitochondrial fragmentation [23, 24]. However, OPA1 cleavage and its function under the pathological condition of neuronal ischemia have not been studied in detail. In this study, we focused on the shear change in OPA1 under the oxygen-glucose deprivation (OGD) model in neurons, and further explored its role in ischemia-reperfusion injury (Fig. S1). Based on several experiments, we reported that ischemia induced neuronal mitochondrial fragmentation accompanied by OMA1-mediated OPA1 cleavage at the S1 site. We also demonstrated that exogenous overexpression of S1-OPA1 exacerbated neuronal mitochondrial fragmentation and dysfunction induced by ischemia and reperfusion. In both cell and animal models, exogenous overexpression of S1-OPA1 exacerbates neuronal reperfusion injury in a GTPase-dependent manner.

## Results

### OGD-induced mitochondrial fragmentation in neurons

**Fig. 1:**
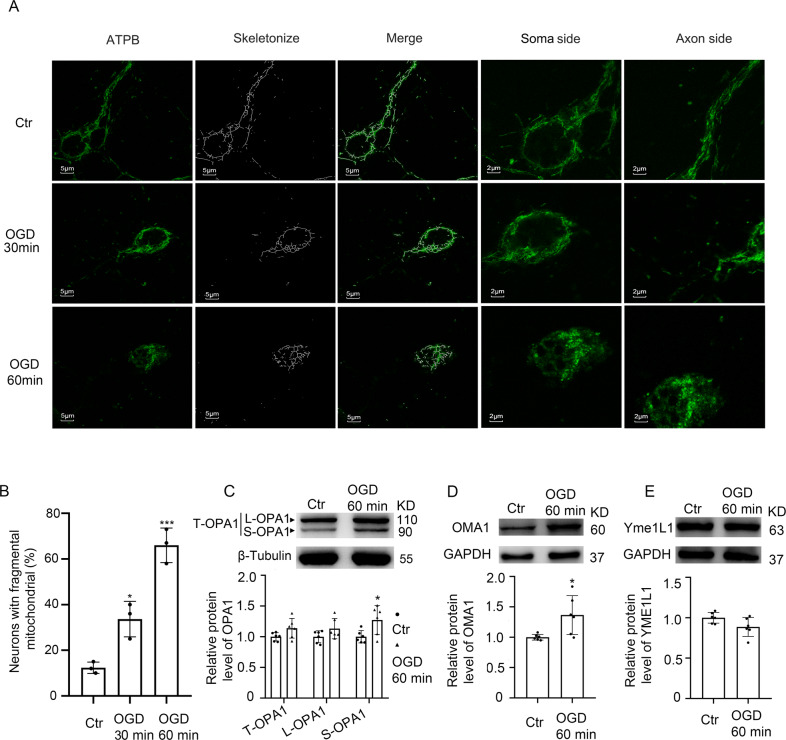
Changes in neuronal mitochondrial networks and OPA1 protein cleavage after OGD. **A** Representative confocal images of mitochondrial networks under normal condition and OGD injury at different times. **B** Representative quantitative results of the percentage of neurons with fragmented mitochondria. Approximately 70–80 cells per experiment were scored. Neuron with fragmented mitochondria: the majority of mitochondria in the neuron displayed a short, fragmented shape, with a length shorter than 20 pixels. ***p < 0.001 vs. control group, *p < 0.05 vs. control group; Scale bar = 5 μm; *n* = 3. **C** Western blot analysis and quantification of long and short OPA1 levels in cultured neurons. **D**, **E** Western blot analysis and quantification of OMA1 and YME1L1 levels in cultured neurons. *p < 0.05 vs. control group; *n* = 6.

To investigate the effect of ischemia on mitochondrial dynamics of neurons, primary cultured neurons were subjected to oxygen-glucose deprivation. Immunofluorescence staining of mitochondrial marker ATPB was performed to analyze the mitochondrial network. In normal cultured neurons, mitochondria were mainly tubular and reticular. After OGD for 30 min, mitochondrial network loss and fragmentation occurred in axons of neurons. When ischemia continued for 60 min, mitochondrial fragmentation also occurred in the neuronal soma (Fig. 1A, B).

### OGD enhanced OMA1-induced OPA1 cleavage at S1 site

To explore the underlying mechanism of mitochondrial fragmentation induced by OGD, we examined the effects of OGD on OPA1, the key regulatory protein of mitochondrial dynamics. After OGD, the total protein level of OPA1 did not significantly increase, but the sheared S-OPA1 protein significantly increased (Fig. 1C). In addition, protein levels of two OPA1 splicing enzymes, OMA1 and YME1L1, were tested. The results indicated that after 60 min of OGD, the protein level of OMA1 significantly increased, while the protein level of YME1L1 did not significantly change (Fig. 1D, E). These results suggested that under OGD, OMA1-induced OPA1 cleavage at S1 site was increased.

### S1-OPA1 overexpression exacerbated mitochondrial fragmentation in neurons exposed to OGD

**Fig. 2:**
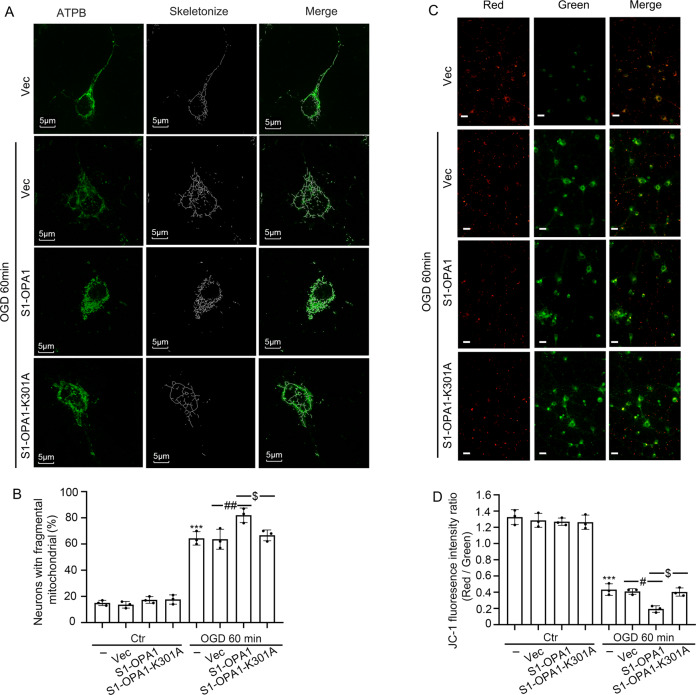
S1-OPA1 overexpression exacerbated mitochondrial fragmentation and membrane potential damage after OGD. **A** Representative confocal images of mitochondrial morphologies in different overexpression groups under normal condition and OGD for 60 min. Scale bar = 5 μm. **B** Representative quantitative results of the percentage of neurons with fragmented mitochondria. **C** Representative images of JC-1 fluorescence staining in different overexpression groups under normal condition and OGD for 60 min. Scale bar = 20 μm. **D** Red/green fluorescence ratio was used to indicate reduced mitochondrial membrane potential. ***p < 0.001 vs. control group, ^##^*p* < 0.01, ^#^*p* < 0.05, ^$^*p* < 0.05, *n* = 3.

To further explore the physiological and pathological effects of increased S1-OPA1 after OGD, we overexpressed S1-OPA1 in primary neurons and observed the effect of this intervention on mitochondrial dynamics in normal and OGD-treated neurons. Considering that S1-OPA1 contains the GTPase site, we also performed exogenous overexpression of the GTPase site mutant, S1-OPA1-K301A. Immunofluorescence and western blot were used to verify the transfection efficiency; the results showed similar transfection efficiency in each group (Fig. S2A–D). Mitochondrial network observed by ATPB immunofluorescence staining showed that the exogenous overexpression had no significant effect on the mitochondrial dynamics of neurons under normal culture conditions, but S1-OPA1 overexpression aggravated mitochondrial fragmentation in neurons exposed to OGD for 60 min. This effect was eliminated by GTPase site mutation (Fig. 2A, B; Fig. S3A). S1-OPA1 did not affect the mitochondrial network of normal neurons, but aggravated the fragmentation of neurons under OGD, suggesting that S1-OPA may interfere with the fusion of ischemia-induced mitochondrial division in a GTPase-dependent manner.

Since the maintenance of mitochondrial membrane potential is closely related to mitochondrial morphology [25], we evaluated the effect of S1-OPA1 overexpression on mitochondrial membrane potential in primary neurons, using the JC-1 kit. We found that S1-OPA1 overexpression had no significant effect on mitochondrial membrane potential in normal cultured neurons, but enhanced OGD-induced mitochondrial membrane potential decline in neurons. However, S1-OPA1-K301A had no significant effect on mitochondrial membrane potential in either normal neurons or OGD neurons (Fig. 2C, D; Fig. S3B).

### S1-OPA1 overexpression exacerbated the destruction of mitochondrial structure in neurons exposed to OGD

**Fig. 3:**
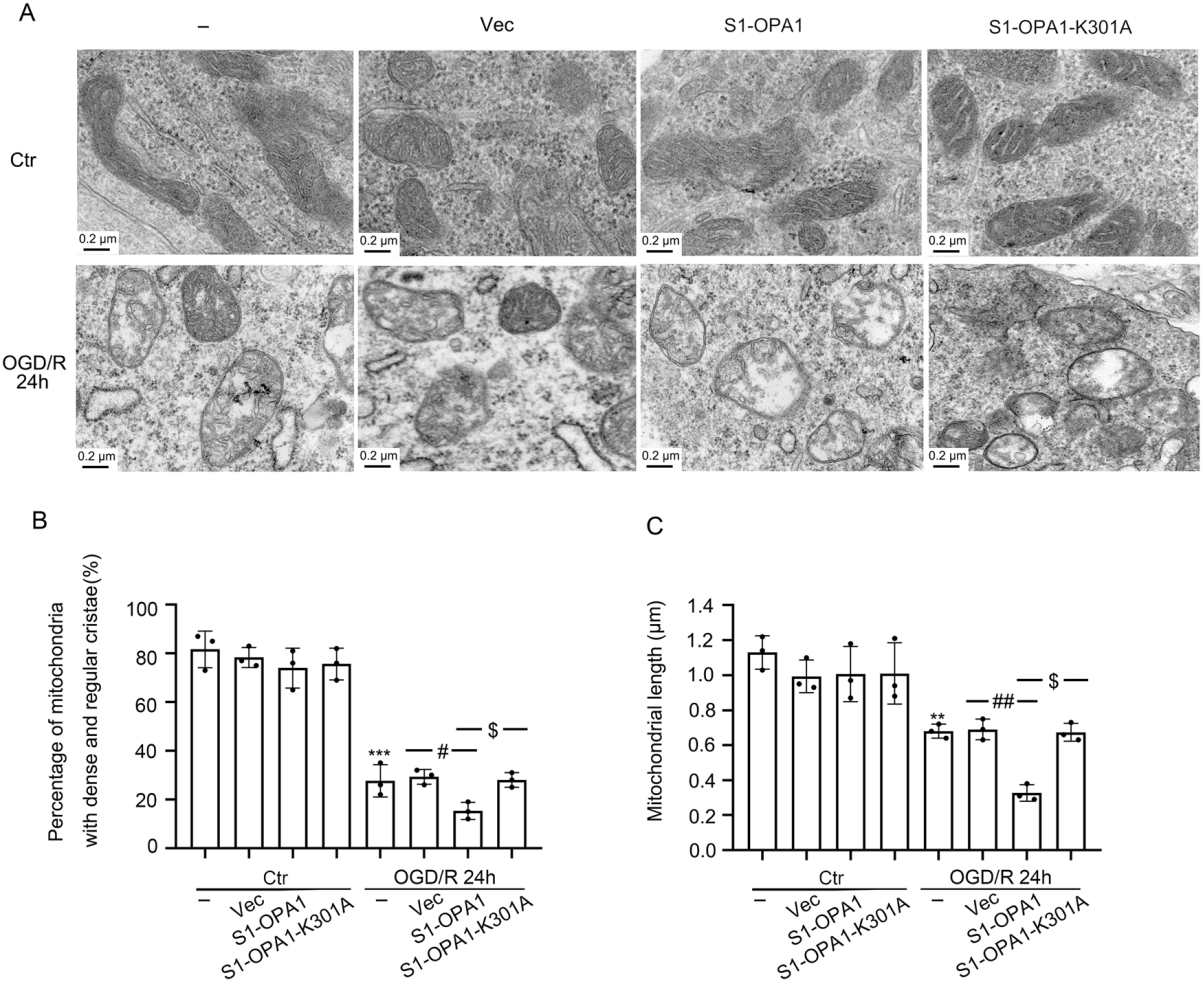
S1-OPA1 overexpression impaired the morphology of mitochondrial cristae and mitochondrial length after OGD/R. **A** Representative transmission electron microscopy images of neuronal mitochondria. Mitochondria in the control group had dense cristae and appropriate mitochondrial length, but after OGD/R for 24 h, the cristae were damaged, mitochondrial edema appeared, and the length was shortened. **B** Fifty mitochondria were selected from each group to assess the proportion of mitochondria with fine and dense cristae. **C** Fifty mitochondria were selected from each group to assess mitochondrial length. ***p < 0.001 vs. control group, **p < 0.01 vs. control group, ^##^*p* < 0.01, ^#^*p* < 0.05, ^$^*p* < 0.05; scale bar = 0.2 μm; *n* = 3.

To further evaluate the effect of S1-OPA1 on the mitochondrial ultrastructure of neurons after OGD, we performed electron microscopy to observe the mitochondrial cristae structure and length in neurons. Dense cristae and appropriate length of mitochondria were the structural basis of the normal mitochondrial function. As shown in Fig. 3A, mitochondrial cristae were damaged after OGD, and the number of mitochondria with dense cristae structure was significantly reduced, accompanied by mitochondrial edema. Meanwhile, S1-OPA1 overexpression significantly exacerbated the cristae injuries, while S1-OPA1-K301 overexpression had no effect (Fig. 3B).

We also measured the mitochondrial length. The results showed that neither the overexpression of S1-OPA1 nor of S1-OPA1-K301A affected mitochondrial length in the control group. OGD resulted in a significant reduction in the mean length of neuronal mitochondria, which was further enhanced by the overexpression of S1-OPA1 but not S1-OPA1-K301A (Fig. 3C).

### S1-OPA1 overexpression exacerbated mitochondrial dysfunction and mitochondrial apoptosis in neurons exposed to OGD/R

**Fig. 4:**
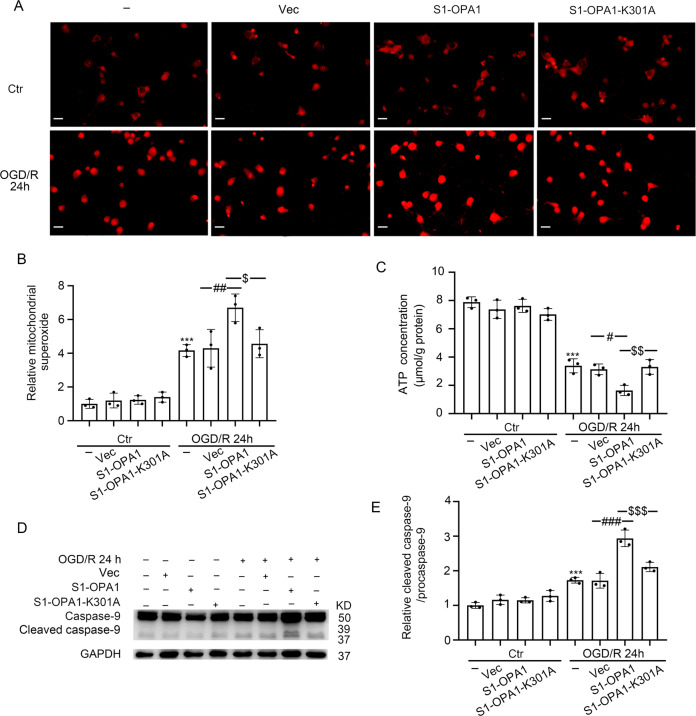
S1-OPA1 overexpression induced mitochondrial superoxide generation, mitochondrial bioenergetic deficits, and mitochondrial apoptosis after OGD/R. **A** Representative images of Mito-Sox staining in different overexpression groups under normal condition and OGD/R for 24 h. **B** Quantitative analysis of superoxide generation in neurons through red fluorescence intensity. **C** ATP content in neurons of each group was leveled by protein concentration. **D** Western blot analysis of caspase-9 and cleaved caspase-9 in neurons. **E** Ratio of cleaved caspase-9 to caspase-9 was quantitatively analyzed to represent the level of mitochondrial apoptosis. ***p < 0.001 vs. control group, ^###^*p* < 0.001, ^##^*p* < 0.01, ^#^*p* < 0.05, ^$$$^*p* < 0.001, ^$$^*p* < 0.01, ^$^*p* < 0.05; scale bar = 20 μm; *n* = 3.

We evaluated the effects of S1-OPA1 overexpression on mitochondrial function in normal and OGD-treated neurons by examining mitochondrial superoxide, bioenergetics, and apoptosis. Neither overexpression of S1-OPA1 nor overexpression of S1-OPA1-K301A affected the level of mitochondrial superoxide or bioenergetics in the control group. When neurons were subjected to OGD/R, the level of superoxidase was significantly increased, and mitochondrial bioenergetics (characterized by ATP content) were significantly reduced, which was further exacerbated by S1-OPA1 overexpression. However, neither the superoxide level nor the ATP content was affected by the overexpression of S1-OPA1-K301A (Fig. 4A–C).

In addition, western blot was performed to detect the protein levels of cleaved caspase-9, an important initiation factor in the mitochondria-dependent apoptosis pathway. The effects of S1-OPA1 overexpression on mitochondria-induced apoptosis in neurons were predicted by cleaved caspase-9 [26]. The results showed that the overexpression of S1-OPA1 did not affect the cleavage of caspase-9 in normal neurons. Overexpression of S1-OPA1 further increased caspase-9 cleavage 24 h after OGD/R, suggesting that S1-OPA1 may induce mitochondria-related apoptosis. Overexpression of S1-OPA1-K301A had no significant effect on cleaved caspase-9 protein level, indicating that the GTPase site played an important role in S1-OPA1 function (Fig. 4D, E).

### S1-OPA1 overexpression intensified neuronal death after ODG/R

**Fig. 5:**
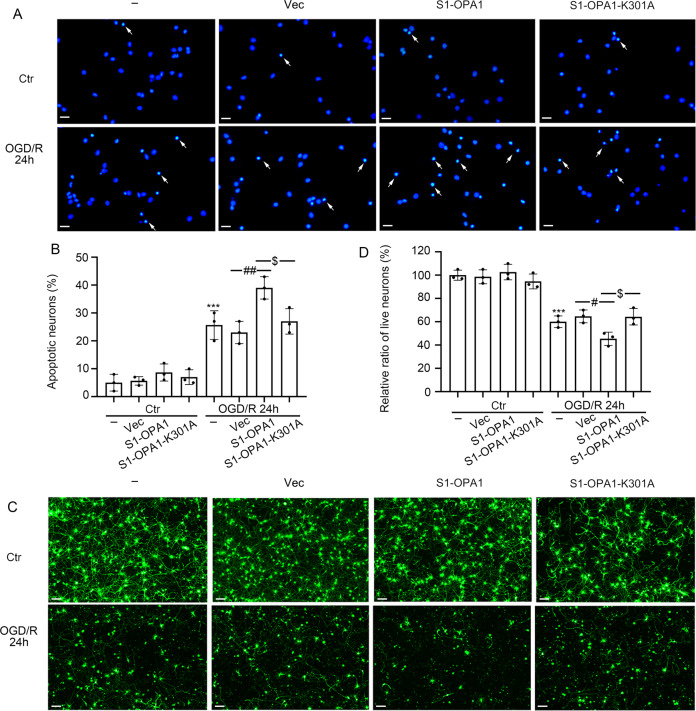
S1-OPA1 overexpression led to neuronal apoptosis and death after ODG/R. **A** Representative images of Hoechst staining in different overexpression groups under normal condition and OGD/R for 24 h. Arrows indicate the nuclei of apoptotic neurons. **B** The percentage of apoptotic neurons was statistically quantified and analyzed. **C** Representative images of live neurons in different overexpression groups under normal condition and OGD/R for 24 h. **D** Quantitative analysis of neuronal viability. ***p < 0.001 vs. control group, ^##^*p* < 0.01, ^#^*p* < 0.05, ^$^*p* < 0.05; scale bar = 20 μm; *n* = 3.

Finally, Hoechst 33258 staining and cell viability assessment were performed to evaluate the effect of S1-OPA1 overexpression on neuronal death. In the control group, neither S1-OPA1 nor S1-OPA1-K301A overexpression caused significant changes in Hoechst positive rate or cell viability. When neurons were subjected to OGD/R, the Hoechst positive rate was significantly increased, and cell viability was significantly reduced, which was further exacerbated by S1-OPA1 overexpression, rather than S1-OPA1-K301A overexpression (Fig. 5A–D).

### MCAO promoted OMA1-induced OPA1 cleavage at S1 site in the brain tissue surrounding ischemia

**Fig. 6:**
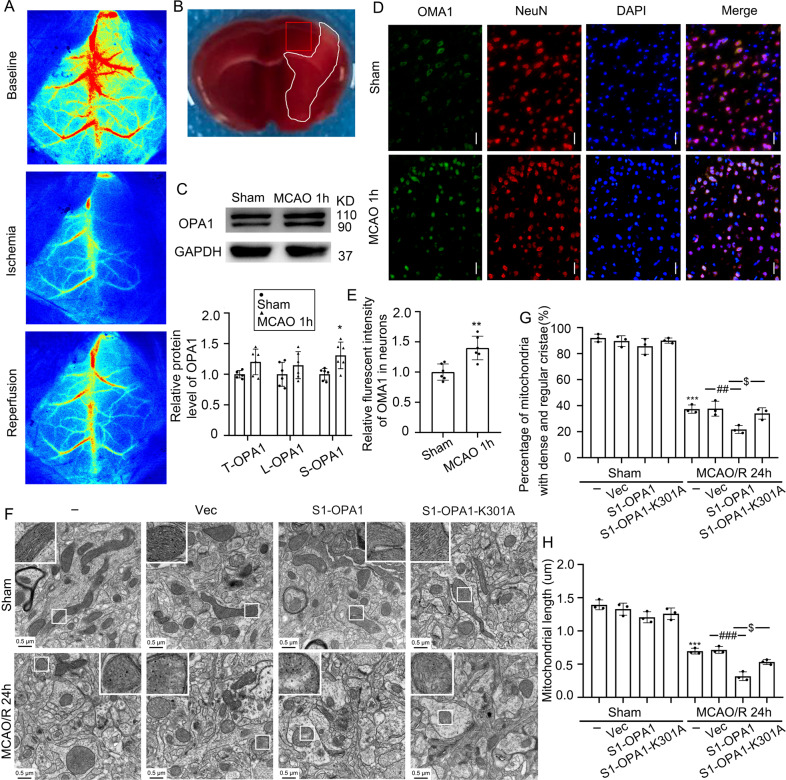
S1-OPA1 was increased in the brain tissue surrounding ischemia and overexpression of S1-OPA1 aggravated mitochondrial ultrastructural damage after MCAO/R. **A** Representative laser speckle images before surgery (baseline), during ischemia, and 10 min after reperfusion. **B** TTC staining 1 h after MCAO in mice. The infarct area is shown in white, and the area of sampling and observation is shown in the red frame. **C** Western blot analysis and quantification of long and short OPA1 levels in brain tissue surrounding ischemia. **D** Double immunofluorescence analysis was performed with anti-OMA1 (green) and neuronal marker (NeuN, red) in brain sections. Nuclei were fluorescently labeled with DAPI (blue). Scale bar = 50 μm; (**E**) The relative fluorescent intensities of OMA1 in neurons were statistically analyzed. **p < 0.01 vs. Sham group; *p < 0.05 vs. Sham group; *n* = 6. **F** Representative transmission electron microscopy images of mitochondria in cortical penumbra. In the sham groups, mitochondria had regular and dense cristae, as well as normal mitochondrial length. In the MCAO/R (24 h) group, the mitochondrial cristae structure was broken. The mitochondrial cristae structure became loose and disorderly, and the length of mitochondria became shorter. Scale bar = 0.5 μm; (**G**) Fifty mitochondria were selected from each group to assess the proportion of mitochondria with dense cristae. **H** Fifty mitochondria were selected from each group to calculate mitochondrial length. ***p < 0.001 vs. Sham group, ^###^*p* < 0.001, ^##^*p* < 0.01, ^$^*p* < 0.05; *n* = 3.

We found that OGD induced OMA1-mediated OPA1 cleavage at the S1 site in in vitro neurons, and S1-OPA1 exacerbated mitochondrial fragmentation and dysfunction, as well as cell death, in neurons subjected to OGD and OGD/R. Based on the above results, we used the mouse MCAO model to further investigate whether S1-OPA1 exists in vivo and changes to S1-OPA1 after ischemia. We used laser speckle to record rCBF levels before surgery, during ischemia, and 10 min after reperfusion to maintain the stability of the MCAO/R model (Fig. 6A and Fig. S4A). In addition, to clarify the ischemic brain region of MCAO mice, we sacrificed the mice 1 h after the establishment of the MCAO model, and created 1-mm brain slices in front of the bregma for TTC staining [27] (Fig. 6B). The levels of OPA1, OMA1, and YME1L1 in the brain tissue surrounding the ischemia were detected by western blot and immunofluorescence staining in the adjacent section of TTC staining. Compared with the sham group, there was no significant increase in the total OPA1 in the brain tissue surrounding the ischemia, but the sheared S-OPA1 protein significantly increased (Fig. 6C). Furthermore, immunofluorescence staining showed that, compared to the sham group, the OMA1 protein level significantly increased in neurons, while the YME1L1 protein level did not significantly change. These results suggested that MCAO induced OMA1-mediated OPA1 cleavage at the S1 site in neurons (Fig. 6D, E and Fig. S4B, C).

### Overexpression of S1-OPA1 worsened mitochondrial structural damage in the brain tissue surrounding ischemia

After identifying the production of S1-OPA1 in the brain tissue surrounding ischemia, we administered AAV9-hSyn-S1-OPA1-mCherry, AAV9-hSyn-S1-OPA1-K301A-mCherry, or control AAV9 into the brain area around the ischemia in mice. As shown in Figure S5A, red fluorescence was observed in the AAV injection area. We further verified the efficiency and specificity of transfection by immunofluorescence and western blot in vivo. First, exogenous cherry was expressed only in NeuN-positive cells, suggesting that AAV9-hSyn-S1-OPA1-mCherry and AAV9-hSyn-S1-OPA1-K301A-mCherry were specifically expressed in neurons (Fig. S5B). Western blot showed that the expression levels of exogenous overexpression were similar in each group, and the MCAO/R model had no effect on exogenous overexpression (Fig. S5C, D).

We used transmission electron microscopy to observe the effect of S1-OPA1 or S1-OPA1-K301A overexpression on mitochondrial ultrastructure in neurons surrounding the ischemia. Twenty-four hours after MCAO/R in mice, we observed that the mitochondrial cristae of neurons were damaged around the ischemia, and the dense mitochondrial cristae structure became loose and chaotic, which was aggravated by S1-OPA1 overexpression rather than by S1-OPA1-K301A (Fig. 6F–G). In addition, compared to the sham group, mitochondrial length of neurons surrounding ischemia was significantly shortened after MCAO/R. Overexpression of S1-OPA1, but not S1-OPA1-K301A, further reduced mitochondrial length in neurons surrounding ischemia in MCAO/R mice (Fig. 6H). In contrast, in sham mice, neither S1-OPA1 nor S1-OPA1-K301A overexpression had an effect on mitochondrial ultrastructure.

### Overexpression of S1-OPA1 increased neuronal degeneration and aggravated cerebral edema, infarction and sensorimotor deficits in MCAO/R mice

**Fig. 7:**
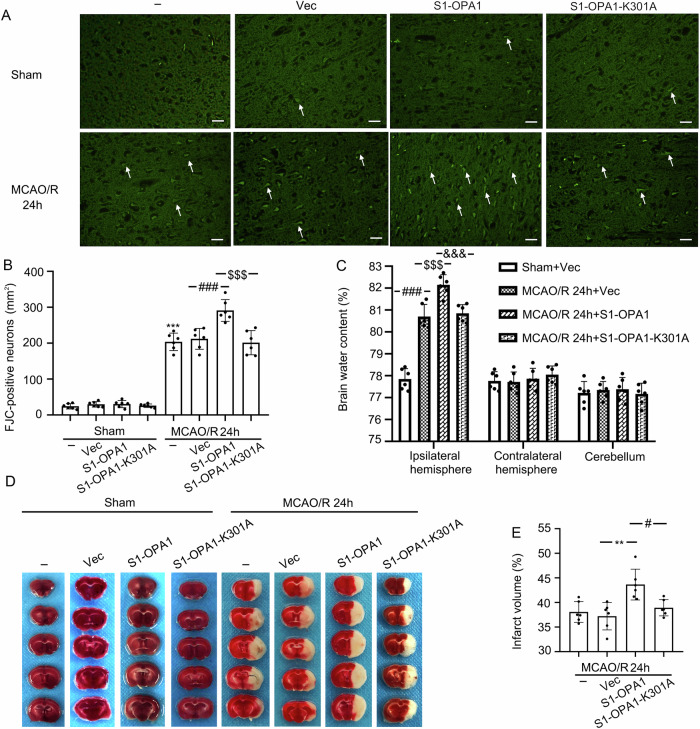
Overexpression of S1-OPA1 increased neuronal degeneration and aggravated cerebral edema and cerebral infarction after MCAO/R. **A** Representative images of FJC staining in sham groups and MCAO/R (24 h) groups. **B** FJC-positive neurons per square millimeter were counted and analyzed. **C** Statistical analysis of brain water content in each group after overexpression of AAVs. **D** Typical TTC stained-images in the sham groups and MCAO/R groups. The light white part of the brain sections indicates the infarct area. **E** The percentage of infarct volume was calculated and statistically analyzed. ***p < 0.001 vs. Sham group, **p < 0.01, ^###^*p* < 0.001, ^#^*p* < 0.05, ^$$$^*p* < 0.001, ^&&&^*p* < 0.001; scale bar = 50 μm; *n* = 6.

To explore the physiological or pathological functions of S1-OPA1 in brains exposed to ischemia-reperfusion, we used FJC staining, brain water content analysis, and TTC staining to detect the effects of S1-OPA and its mutants on peripheral ischemic neuron degeneration, brain edema, and cerebral infarct volume in mice 24 h after MCAO/R modeling. Compared to the sham group, MCAO/R significantly induced neuronal degeneration, brain edema, and cerebral infarction, which were significantly aggravated by S1-OPA1 overexpression. However, no such change was induced by S1-OPA1-K301 overexpression, and neither S1-OPA1 nor S1-OPA1-K301 overexpression had an effect in sham mice (Fig. 7A–E).

### Overexpression of S1-OPA1 aggravated sensorimotor deficits in MCAO/R mice

**Fig. 8:**
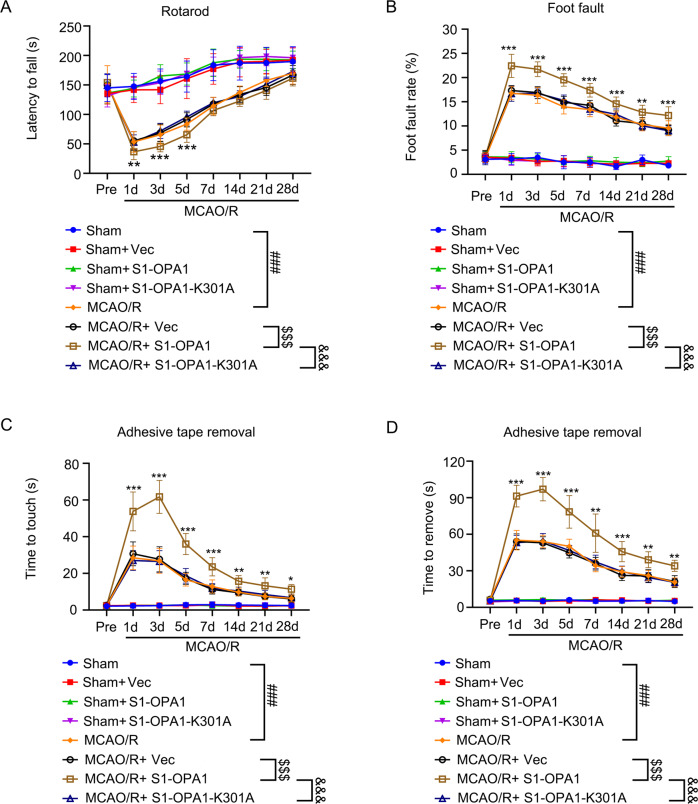
Overexpression of S1-OPA1 aggravated the sensorimotor deficit after MCAO/R. Sensorimotor deficits were evaluated before (pre) and up to 28 days after MCAO/R or sham (**A**) Rotarod test. The time taken by the mice to drop off from the rotating drum was recorded as the latency to fall and statistically analyzed. **B** Foot fault test. The proportion of the foot fault steps of the left upper limb among all steps was recorded and statistically analyzed. **C**, **D** The time to touch and removal of the stickers was recorded and statistically analyzed. ***p < 0.001 MCAO/R + Vec vs. MCAO/R + S1-OPA1, **p < 0.01 MCAO/R + Vec vs. MCAO/R + S1-OPA1, *p < 0.05 MCAO/R + Vec vs. MCAO/R + S1-OPA1, ^###^*p* < 0.001, ^$$$^*p* < 0.001, ^&&&^*p* < 0.001; *n* = 12.

Finally, rotarod test, foot fault test, and adhesive removal test were performed to evaluate sensorimotor deficits in MCAO/R mice, and to explore whether S1-OPA1 was involved. Rotarod test showed that on days 1, 3, and 5 after MCAO/R, the time on the rotarod in the S1-OPA1 overexpression group was shorter than that in the Vec group, suggesting that the mice in the S1-OPA1 overexpression group had weaker motor coordination. However, there was no reduction in the latency time in the S1-OPA1-K301A group compared to the Vec group (Fig. 8A). The foot fault test (Fig. 8B) showed that 1–28 days after MCAO/R, the rate of foot fault steps in the S1-OPA1 group was higher than that in the Vec group, indicating heavier damage in the S1-OPA1 group. However, there was no significant change in the S1-OPA1-K301A group. Finally, the adhesive removal test showed that overexpression of S1-OPA1 significantly prolonged the time for mice to touch and remove the sticker after MCAO/R. However, compared to the Vec group, the touch and removal time was not increased in the S1-OPA1-K301 group. In the sham group, neither the overexpression of S1-OPA1 nor of S1-OPA1-K301A affected sensorimotor function (Fig. 8C, D).

As shown above, we found that exogenous overexpression of S1-OPA1 not only aggravates ischemia-induced mitochondrial fragmentation, but also exacerbates reperfusion brain injury. In addition to losing its role in promoting mitochondrial fusion, the mechanism by which S1-OPA1 aggravates neuronal ischemia-reperfusion injury remains to be further studied. We observed proteins interacting with OPA1 using STRING (Functional Protein Association Network) system and found that Drp1 has the potential to interact with OPA1 (Fig. S6A). Therefore, we investigated Drp1 protein level and phosphorylation level after OGD injury. We found that Drp1 protein level increased 60 min after OGD treatment (Fig. S6B), and the phosphorylation of Drp1 at Ser616 was significantly increased compared with that at Ser637 (Fig. S6C, D). These results indicate that more Drp1 can be recruited to the outer membrane of mitochondria to promote fission under OGD injury, and whether OPA1 or S1-OPA1 is involved in this process arouses our concern. However, co-IP experiments were performed and no interaction between OPA1 and Drp1 was found (Fig. S6E).

## Discussion

As a key mechanism of mitochondrial quality control, mitochondrial dynamics have become an important target of neuronal protection after cerebral ischemia-reperfusion [28]. Previous studies have focused on excessive mitochondrial fission in neurons after reperfusion [29]. Neuronal mitochondrial fission is induced by ischemia. However, the effect of this process on mitochondrial dynamics during subsequent reperfusion periods is not well understood. Sophisticated regulation of mitochondrial dynamics plays a key role in maintaining mitochondrial function and cell homeostasis in neurons [30, 31]. Mitochondrial fusion is an evolutionary conservative process mediated by three large GTPases from the dynein superfamily. OPA1 is a large GTPase, which mediates mitochondrial inner membrane fusion [32]. Since S1-OPA1 contains the GTPase site, site-directed mutation and mutant overexpression were performed, and we found that S1-OPA1 exacerbated mitochondrial fragmentation mediated by ischemia and reperfusion in a GTPase-dependent manner. In addition, Langer et al. found co-localization of OPA1 and Drp1 in mitochondria through immunofluorescence in mouse embryonic fibroblasts [23]. However, Drp1 is located in the cytoplasm or mitochondrial outer membrane, while OPA1 is distributed in the mitochondrial inner membrane and matrix. Owing to the different spatial distribution of Drp1 and OPA1, co-localization or direct interaction cannot be achieved. Moreover, Langer in his subsequent review mentioned the uncertainty surrounding the relationship between OPA1 and Drp1 [33]. However, we found no substantial evidence of the interaction between S1-OPA1 and DRP1 in this study. Therefore, one limitation of this study was that we only found the mitochondrial injury effect of S1-OPA1 after ischemia. We did not thoroughly search for the molecular mechanism of mitochondrial fragmentation and the corresponding injury. However, we will attempt to explore these mechanisms in future studies.

In various disease models, such as heart failure, kidney ischemia, or neurodegenerative diseases, OMA1-mediated cleavage of OPA1 at the S1 site does not cause mitochondrial fusion. However, it may cause mitochondrial fragmentation or even mitochondrial apoptosis. Therefore, knockout or knockdown of OMA1 has become a common treatment strategy. Studies have also reported the effectiveness of this mitochondrial protective measure [34–36]. Another treatment strategy may be to express OPA1, which will strengthen the function of mitochondrial fusion, and compensate for lack of mitochondrial fusion caused by increased shear at S1 sites, thereby reducing mitochondrial fragmentation [37, 38]. Moreover, some studies overexpressed L-OPA1 with deletion of the S1 site. In this case, only fusion function of L-OPA1 is available, and experimental results show effective protective effects of mitochondria [39, 40]. However, different opinions have been stated. One study reported that the existence of only S1-OPA1 would maintain the cristae structure and respiratory function of mitochondria, and that this function depends on the GTP activity of S1-OPA1, which is in contrast to most other study results [41]. However, this study did not use a disease model. This was a separate study of the role of S1-OPA1 under the condition with internal OPA1 clearance, which is different from the pathological conditions in most studies. This may explain the differences in study results between this and previous studies. In our study, we also found that overexpression of S1-OPA1 under normal circumstances did not cause mitochondrial damage.

In this study, we constructed an in vitro OGD model and used a confocal microscope to observe the network structure and fragmentation of neuronal mitochondria. However, mitochondrial network structure is difficult to observe in in vivo MCAO models. However, in the in vivo experiments, we verified that S1-OPA1 overexpression can regulate mitochondrial morphology through transmission electron microscopy, and also observed the effect of S1-OPA1 on ischemia-reperfusion injury. This is the first step of translational research because cultured neurons, which performed OGD damage, cannot completely simulate the ischemia and hypoxia in vivo. In the MCAO/R model, there are neurovascular [42] and neuron-glial [43] networks, as well as complex interactions between various cells, making the results difficult to analyze. Clinical application of basic research is problematic because of differences between in vivo and in vitro experiments, and between species.

In general, under normal circumstances, OMA1 and YME1L1, as the two cleavage enzymes of OPA1, maintain a certain balance, so that the protein cleavage of the S1 and S2 sites of OPA1 maintains a balance, thereby regulating the normal fusion of neuronal mitochondria. However, when neurons suffer from ischemic stroke, the protein level of OMA1 increases, leading to increased OPA1 cleavage at the S1 site mediated by it, and increased S1-OPA1 aggravates ischemia-induced neuronal mitochondrial fragmentation and dysfunction in a GTPase-dependent manner, eventually leading to ischemia-reperfusion injury of neurons (Fig. S7).

## Materials and methods

### Ethics and animals

All experiments were approved by the Institutional Animal Care and Use Committee of the First Affiliated Hospital of Soochow University, Suzhou, Jiangsu Province. The study was performed in accordance with the Guide for the Care and Use of Laboratory Animals of the National Institutes of Health, and reported in compliance with the ARRIVE (Animal Research: Reporting of In Vivo Experiments) guidelines. Male adult C57BL/6 mice (weight: 25–30 g; Animal Center of Chinese Academy of Sciences, Shanghai, China) were housed at a relatively constant temperature (20–26 °C) and humidity (40–70%), under 12-h light/dark cycle. Food and water were supplied without restriction. All experimental animals were coded consecutively, and then we used an online random number generator to generate a random order table. The animals were grouped according to a random order table. Experiment performers and experiment data collectors were unaware of the experimental groupings. If animals died due to modeling and other reasons in the experiment, we supplemented them according to the random order table.

### Establishment of transient middle cerebral artery occlusion/reperfusion (tMCAO/R) in C57BL/6 mice

Transient focal cerebral ischemia was simulated in adult male mice by intracavitary occlusion of the right middle cerebral artery (MCA) for 60 min, followed by recanalization [44]. Mice were anesthetized with 3% isoflurane using small animal gas anesthesia machine (R500IP; RWD Life Technology Co., Shenzhen, China), and anesthesia was maintained with 1.5% isoflurane. The distal end of the external carotid artery (ECA) was cut open, and a monofilament was inserted along the ECA into the internal carotid artery (ICA). After reaching the MCA origin, the artery was embolized for 60 min, followed by removal of the monofilament. Mice were placed in a thermostatic blanket to maintain a constant anal temperature throughout the procedure. Regional cerebral blood flow (rCBF) was monitored before ischemia, 10 min after MCAO, and 10 min after reperfusion, using laser speckle imaging system (RFLSIIII, RWD Life Technology Co.). Mice that did not show a rCBF reduction of at least 75% from the baseline levels after embolization were excluded from further experiments. Mice in the sham operation group were anesthetized in the same way, and the distal ECA was ligated.

### Primary neuron culture

Based on our previous experience [45], primary cortical neurons were extracted from 16-day-old mouse embryos. First, the whole brains of fetal mice were extracted, and the cerebellum and brainstem were removed. Only the bilateral cortex was left in the pre-cooled PBS. The blood vessels and arachnoids were microscopically removed from the surface of the bilateral cortex. Next, the cortical tissue was digested using 0.25% trypsin at 37 °C for 5 min, washed with PBS three times, and blown into the cell suspension. The suspension was centrifuged at 500 × g for 5 min. The dissociated neurons were plated at a density of 1 × 10^6^ cells/well on a six-well plate, and at 2 × 10^5^ cells/well on a 12-well plate (Corning, USA), coated with poly-D-Lysine (Thermo Fisher Scientific, Waltham, MA, USA). The neuron culture medium included Neurobasal Medium, B27 supplement, 0.5 mM glutamine, and penicillin/streptomycin (Thermo Fisher Scientific). The culture environment was maintained at 37 °C with humidified 95% air and 5% CO_2_.

### Establishment of oxygen-glucose deprivation/reoxygenation (OGD/R) model

As previously reported [46], complete neuron culture medium was replaced with Neurobasal Medium-no D-glucose (Thermo Fisher Scientific) and neurons were transferred to a 5% CO_2_ and 95% N_2_ atmospheric incubator for 1 h at 37 °C. Then, neurons were cultured in complete neuron culture medium at 37 °C under humidified 95% air and 5% CO_2_. The control group was cultured according to the routine procedures during this period.

### Antibodies

Mouse monoclonal anti-ATPB antibody (ab14730), rabbit polyclonal anti-OPA1 antibody (ab42364), rabbit polyclonal anti-OMA1 antibody (ab154949), anti-NeuN antibody (ab177487), and rabbit monoclonal anti-caspase 9 antibody (ab184786) were purchased from Abcam (Cambridge, UK). Rabbit polyclonal anti-YME1L1 antibody (11510-1-AP) was purchased from Proteintech (Wuhan, China). Alexa Fluor-488 donkey anti-mouse IgG antibody (A21202) was obtained from Life Technologies (Carlsbad, CA, USA) as secondary antibodies for immunofluorescence. Mouse monoclonal anti-Flag antibody (8146), rabbit monoclonal anti-GAPDH antibody (5174), rabbit monoclonal anti-β-tubulin antibody (2128), anti-rabbit-IgG-HRP (7074), and anti-Mouse-IgG-HRP (7076) were purchased from Cell Signaling Technology (Berkeley, CA, USA). Specific information about antibodies was presented in Table S1.

### Immunofluorescent analysis

As previously mentioned [47], in vitro cultured primary cortical neurons were fixed with 4% paraformaldehyde for 30 min and washed with PBS three times. Then, the neurons were blocked with 5% BSA for 30 min, and incubated overnight at 4 °C with primary antibodies (diluted 1:300) and for 1 h at 37 °C with secondary antibodies (diluted 1:300). Finally, the neurons were observed with Stimulated Emission Depletion (STED) microscope (Suzhou Institute of Biomedical Engineering and Technology, Chinese Academy of Sciences, Beijing, China) and fluorescent microscope (OLYMPUS BX50/BX-FLA/DP70; Olympus Life Science Europa GMBH, Hamburg, Germany). The in vivo brain tissue was cut into 10-μm frozen sections, and the antigens were repaired using antigen retrieval solution for 20 min. Next, 0.1% Triton was added for 10 min, and 5% BSA was used to block these sections for 1 h at room temperature. The target protein primary antibody was added, and the solution was incubated overnight at 4 °C. Then, the corresponding fluorescent secondary antibody was added and the solution was incubated at 37 °C for 1.5 h. Brain sections were sealed with DAPI and observed under a fluorescence microscope.

### Western blot analysis

Based on previous studies [48], the cultured primary cortical neurons and brain tissue surrounding the infarct were collected and lysed in Western lysis buffer (Beyotime Biotechnology Inc., Shanghai, China) on ice for 20 min. After centrifugation at 12000 r/min for 10 min, the supernatant was used to detect the protein concentration with an enhanced BCA Protein Assay Kit (Beyotime Biotechnology Inc.). Then, the protein samples (30 μg/lane) were loaded onto 10% SDS polyacrylamide gel for electrophoretic separation, and then transferred to a polyvinylidene difluoride (PVDF) membrane (IPVH00010; Millipore, Billerica, MA, USA). The membrane was immediately blocked with 5% bovine serum albumin (Biosharp, Hefei, China) for 1 h at room temperature. Then, the membrane was incubated overnight with the primary antibody at 4 °C, and with the HRP-conjugated secondary antibodies for 1 h at room temperature. Finally, the membrane was revealed with an enhanced chemiluminescence (ECL) kit (Beyotime Biotechnology Inc.). Additionally, β-tubulin and GAPDH were used as loading controls. Full length uncropped original western blots were shown in Supplementary materials 2.

### Transduction of lentivirus and adeno-associated virus

We constructed three in vitro lentiviruses containing the mitochondrial targeting signal (LV-Flag, LV-S1-OPA1, LV-S1-OPA1-K301A; GeneChem, Shanghai, China). The sequence elements of the lentiviral vector were Ubi-MCS-3FLAG-SV40-puromycin. The titers of LV-Flag and LV-S1-OPA1 WT were 3 × 10^8^ TU/mL, and the titer of LV-S1-OPA1-K301A was 2 × 10^8^ TU/mL. After four days of extraction and culture, primary neurons were transfected with the corresponding LV using 20 μl HitransGA (GeneChem) to enhance the transduction efficiency. We calculated the virus usage according to the following formula: virus volume = MOI * cell count/virus titer (multiplicity of infection [MOI] = 10). Three days after the transduction, neurons were exposed to OGD.

Corresponding to the in vitro experiments, three adeno-associated viruses (AAVs) were constructed in vivo to regulate S1-OPA1 in in vivo neurons (AAV9-Flag, AAV9-S1-OPA1, and AAV9-S1-OPA1-K301A; GeneChem). The sequence elements of the AAV vector were hSyn promoter-MCS-cherry-3FLAG-SV40 PolyA. We selected the AAV9 serotype and the neuron-specific promoter hSyn to achieve accurate transfection of neurons in vivo [49]. Localized injection was performed in the cortical region of the penumbra (AP: +0.3 mm, 0.8 mm, and 1.9 mm; ML: 2.5 mm; DV: −2 mm), and the total amount of AAV used was 2E + 10 v.g. The titer of the AAV9-Flag and AAV9-S1-OPA1 was 2E + 13 v.g./mL, and the titer of the AAV9-S1-OPA1-K301A was 1.77E + 13 v.g./mL. The transfection efficiency was verified three weeks after localized injection, and subsequent experiments were conducted. Specific information about S1-OPA1 gene regulation was shown in Table S2.

### Detection of mitochondrial membrane potential

The mitochondrial membrane potential of primary neurons was detected using the mitochondrial membrane potential detection kit (JC-1; Beyotime Biotechnology Inc.). JC-1 is an ideal fluorescent probe widely used to detect mitochondrial membrane potential [50]. When the mitochondrial membrane potential is high, JC-1 aggregates in the mitochondrial matrix to form J-aggregates, and generates red fluorescence. When the mitochondrial membrane potential is low, JC-1 cannot accumulate in the mitochondrial matrix. Therefore, JC-1 remains monomeric and produces green fluorescence. Dye solution was diluted with dye buffer and added to the culture plate for staining at 37 °C for 20 min. Neurons were washed twice with dye buffer and then observed under fluorescence microscope (OLYMPUS BX50/BX-FLA/DP70; Olympus).

### Transmission electron microscopy

The in vitro adherent neurons were gently scraped with cell paddle and centrifuged at 1000 rpm for 5 min. The supernatant was discarded, and the cell masses were fixed with 2.5% glutaraldehyde fixation solution. In vivo, the 1-mm^3^ brain tissue surrounding the infarction was quickly removed within 1 min of the sacrifice of the mice, and placed in 2.5% glutaraldehyde for fixation. After fixation, these samples were washed with 0.1 M phosphoric acid rinse solution. The samples were dehydrated in 50% ethanol, 70% ethanol, 90% ethanol, 100% ethanol, and 100% propylene oxide. After embedding with propylene oxide + embedding solution (2:1), 1-μm sections were created with an ultra-thin microtome. The sections were double stained with 3% uranyl acetate-lead citrate, and observed by transmission electron microscope (HT7700, Hitachi, Tokyo, Japan).

### Measurement of mitochondrial superoxide indicator

Mitochondrial superoxide in primary neurons was detected by MitoSOX™ Red reagent (Thermo Fisher Scientific). MitoSOX™ Red mitochondrial superoxide indicator is a novel fluorogenic dye that is highly selective for the detection of superoxide in the mitochondria of live cells [51]. MitoSOX™ Red reagent is live-cell permeant, and is rapidly and selectively targeted to the mitochondria. Once in the mitochondria, MitoSOX™ Red reagent is oxidized by superoxide and exhibits red fluorescence. The adherent neurons were covered with 1.0 mL of 5 μM MitoSOX™ reagent working solution, and incubated for 10 min at 37 °C. The neurons were washed three times with PBS. Finally, fluorescence microscope was used to observe the neurons, and the intensity of red fluorescence was calculated using ImageJ software (US National Institutes of Health, Bethesda, MD, USA).

### ATP content detection

Enhanced ATP detection kit (Beyotime Biotechnology Inc.) was used to detect ATP content in neurons [52]. In accordance with the manufacturer’s instructions, cells were first lysed with lysis buffer, and centrifuged at 4 °C at 12,000 g for 5 min. Then, 20 μL of the supernatant and 100 μL of ATP detection working solution were mixed. Finally, the luminescence module of the multifunctional microplate reader (Spark 10 M; Tecan Australia, Port Melbourne, VIC, Australia) was used for detection. The ATP concentration in each experimental group was quantified according to the ATP standard curve, and standardized by protein concentration.

### Detection of neuronal apoptosis

Hoechst 33258 staining kit (Beyotime Biotechnology Inc.) was used to detect neuronal apoptosis [53]. After Hoechst staining, the nucleus of normal neurons appeared blue, while the nucleus of apoptotic neurons appeared dense or fragmented with white color. Neurons were fixed with fixative solution for 20 min and washed with PBS three times, followed by staining with dye solution at room temperature for 5 min. After washing with PBS three times, neurons were sealed with anti-fluorescence quenching sealing solution, and observed under fluorescence microscope.

### Live neuron staining

Neuronal viability was evaluated using cell viability kit (Invitrogen, Carlsbad, CA, USA). Calcein AM is a nonpolar dye that dyes living cells and is passively transported and accumulated into cells [54]. Inside the cells, calcein AM, which has almost no fluorescence, is hydrolyzed by intracellular esterase to remove methyl acetyl and generate fluorexon. Fluorexon is a polar fluorescent dye with no membrane permeability, which allows it to remain inside the cells and giving the cytoplasm, including the mitochondria, a strong green fluorescence. The adherent neurons were added to 5 µM calcein AM staining solution, and incubated at room temperature for 40 min, without light exposure. These neurons were washed with PBS three times and observed under fluorescence microscope.

### FJC staining

As in previous studies, FJC staining was used to detect neuronal degeneration [55]. Brain slices were dried at 60 °C for 24 h and eluted in an alcohol gradient. Then, they were immersed in a 0.06% potassium permanganate solution for 10 min, and transferred to a 0.0001% FJC (EMD; Millipore Corp., Milford, MA, USA) solution, dissolved in a 0.1% acetic acid vehicle. The solution was incubated for 10 min. Finally, brain slices were baked at 50 °C for 10 min, mounted with neutral gum, and the stained degenerating neurons were observed under fluorescence microscope.

### Brain water content assessment

Wet/dry method was used to measure brain water content [56]. We removed the brain and cut it into three parts 24 h after MCAO/R: the ipsilateral hemisphere, contralateral hemisphere, and cerebellum. The wet weights of these three parts were measured. Then, the three parts were dried at 100 °C for 24 h, and their dry weight was measured. Brain water content was assessed using the following formula: ([wet weight − dry weight]/wet weight) × 100%.

### 2, 3, 5-Triphenyltetrazolium chloride (TTC) staining

Infarction was evaluated by TTC staining [57] 24 h after MCAO/R. The mouse brain was immediately removed and sliced into 1-mm slices from the frontal pole in the coronal direction. The brain slices were immersed in the TTC dye solution (Jiancheng Biotech, Nanjing, China) and incubated at 37 °C for 15–30 min. A digital camera was used for recording, and Image J software was used to calculate the infarct volume. To correct the effect of brain swelling on the calculation of infarct volume, infarct volume was represented by the area of the contralateral complete hemisphere minus the area of the ipsilateral non-infarcted tissue. The percentage of infarct volume was expressed as the ratio of the sum of infarct areas from all slices of each brain to the total brain area.

### Rotarod test

We used a rotarod fatigue instrument (SA102, SANS Biological Technology Co., China) to perform mice rotarod test. Mice were placed on a rotating drum, which accelerated from 4 to 40 rpm in 2 min. The duration until the mice dropped off from the rotating drum was recorded as the latency time [58]. The preoperative training continued for five days. On the first and second days, the mice were trained on a rotating rod at a low speed (accelerating from 0 to 10 rpm within 30 s) for 5 min, once a day. On the third day, one low-speed and one high-speed (accelerating from 4 to 40 rpm in 2 min) training were successively performed for 5 min. On the fourth day, mice were trained twice at high speed. On the last day of the training, mice were trained three times at high speed, and the data were recorded as the baseline. After the operation, these mice were subjected to high-speed rotarod test 3 times per test day, with an interval of 15 min. The longest time for each test was 5 min. The average value was recorded as the fall latency. Postoperative tests were performed at 1, 3, 5, 7, 10, 14, 21, and 28 days after tMCAO surgery.

### Foot fault test

For the foot fault test [59], we constructed a metal shelf (30 cm [L] × 35 cm [W] × 30 cm [H]), with a 2.25 cm^2^ (1.5 cm × 1.5 cm) grid directly above the shelf. Mice were placed on the grid for 1 min, and the total number of steps of the left upper limb (number of times the left upper limb was raised and lowered on the grid) and the number of foot faults (number of times the left upper limb fell from the grid) were recorded. Left forelimb damage was represented by the percentage of the number of foot fault/total number of steps. The mice received training for 5 consecutive days before the operation, three times a day for 1 min each. The total number of steps and foot fault of the left upper limb were counted by blinded investigators. The training data from the last day before surgery was used as the baseline. The test was performed on days 1, 3, 5, 7, 10, 14, 21, and 28 after surgery.

### Adhesive removal test

As described in the previous studies [60], the same sized tape (0.3 × 0.4 cm^2^) was attached to the inner side of the left radius of the experimental mice with equal pressure. The mice were gently put into a transparent squirrel cage, and the time of touch and removal of the tape was recorded. The longest time was 120 s. The mice were trained for five consecutive days, three times per day, before the operation. The tests were performed at 1, 3, 5, 7, 10, 14, 21, and 28 days after the operation, three times a day, with an interval of 15 min between each test. The average time of the three tests of touching and removing the sticker was calculated.

### Statistical analysis

All data are expressed as mean ± standard deviation (SD). GraphPad Prism 8 (GraphPad Software Inc., San Diego, CA, USA) was used for statistical analysis. Before performing variance analysis, we used the Shapiro–Wilk test to test whether the data conformed to a normal distribution. The comparative analysis between the two groups was performed using the unpaired *t* test. Brown-forsythe test is used to test homogeneity of variance, and variables were compared between the groups using one- or two-way analyses of variance (ANOVA). *P* value < 0.05 was considered statistically significant. Detailed statistics were shown in Table S3.

## Supplementary information

Supplementary materials 1

Supplementary materials 2

Revised manuscript(CDDIS-21-4311R the original revision)

Revised manuscript with marked Revision (CDDIS-21-4311R the original revision)

Reproducibility checklist

## Data Availability

The datasets used and/or analyzed during the current study are available from the corresponding author on reasonable request.
