# Author Correction: Ischemia-induced cleavage of OPA1 at S1 site aggravates mitochondrial fragmentation and reperfusion injury in neurons

**DOI:** 10.1038/s41419-024-07032-7

**Published:** 2024-09-04

**Authors:** Xiang Li, Haiying Li, Zhongmou Xu, Cheng Ma, Tianyi Wang, Wanchun You, Zhengquan Yu, Haitao Shen, Gang Chen

**Affiliations:** 1 Department of Neurosurgery & Brain and Nerve Research Laboratory, The First Affiliated Hospital of Soochow University, 188 Shizi Street, 215006 Suzhou, Jiangsu Province China; 2 Institute of Stroke Research, Soochow University, Suzhou, China

**Keywords:** Stroke, Cell death in the nervous system

Correction to: *Cell Death and Disease* 10.1038/s41419-022-04782-0, published online 08 April 2022

The authors regret that errors occurred during the assembly of Fig. 7. Specifically, the representation of FJC staining for the MCAO/R + Vec group in Fig. 7A is incorrect. The corrected Fig. 7A is shown below. The correction does not impact the results or conclusions of this study.
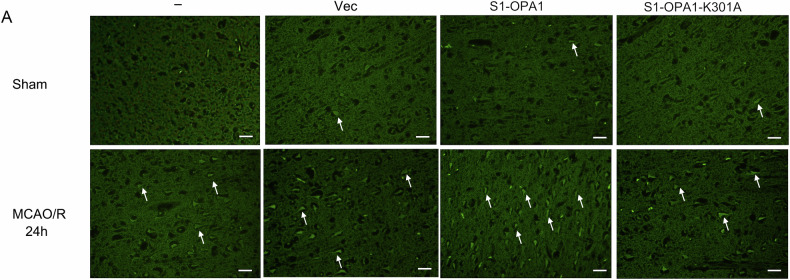


The authors apologize for any inconvenience caused.

The original article has been corrected.

