# Supplementary material for: Ischemia-induced cleavage of OPA1 at S1 site aggravates mitochondrial fragmentation and reperfusion injury in neurons
Source: Cell Death Dis. 2022 Apr 8;13(4):321. doi: 10.1038/s41419-022-04782-0 (PMC8993832; doi:10.1038/s41419-022-04782-0)
Supplement: Supplementary file 1 — Supplementary materials 1 [file 41419_2022_4782_MOESM1_ESM.docx]

**Supplementary materials 1**

**Figure S1**

**
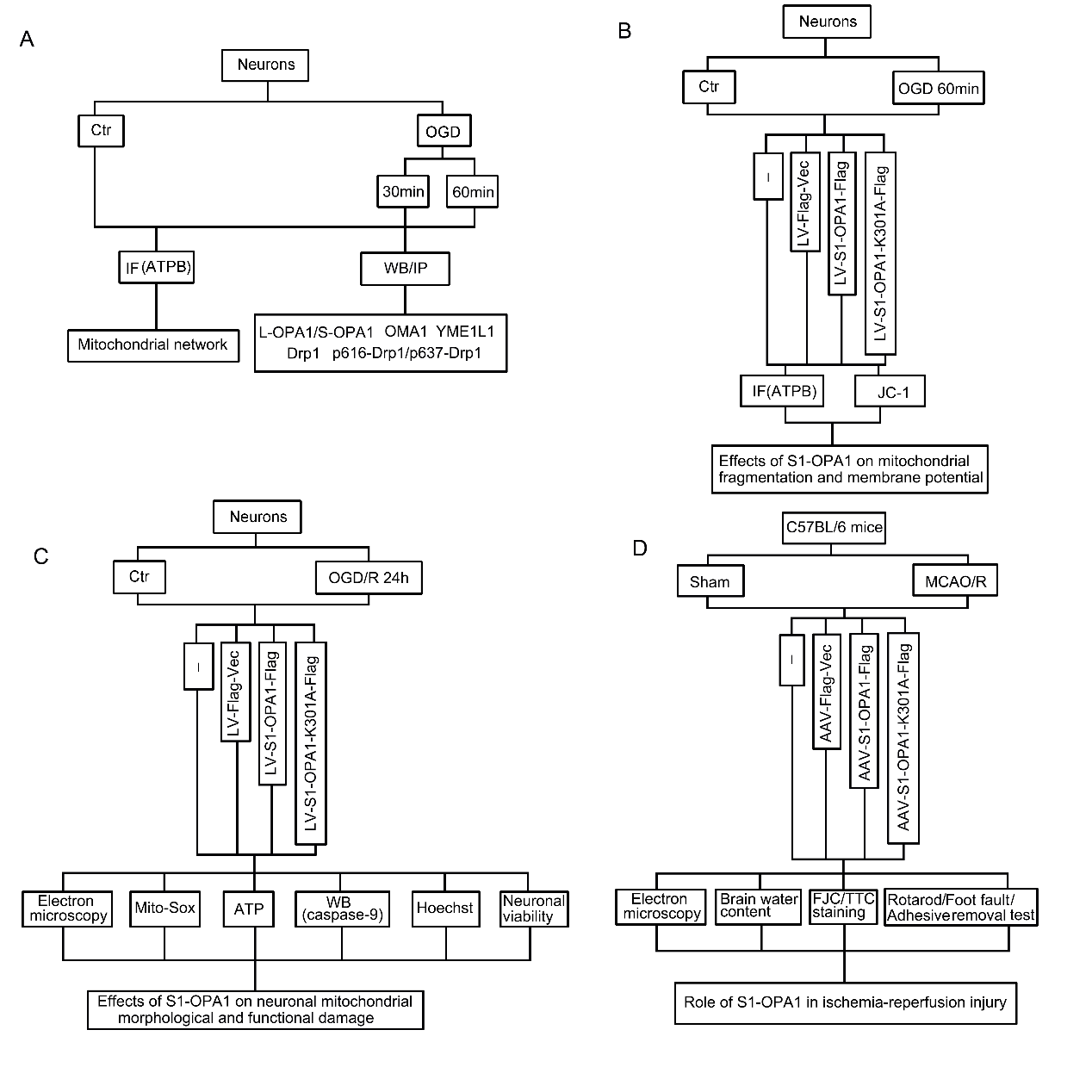
 Figure S1. Experimental groups and study design.** (A) Changes in mitochondrial network and its key regulatory proteins in neurons undergoing OGD. (B) Effects of S1-OPA1 on mitochondrial fragmentation and membrane potential. (C) Effect of S1-OPA1 on mitochondrial morphology and mitochondrial function damage. (D) Role of S1-OPA1 in ischemia-reperfusion injury.

**Figure S2**

**
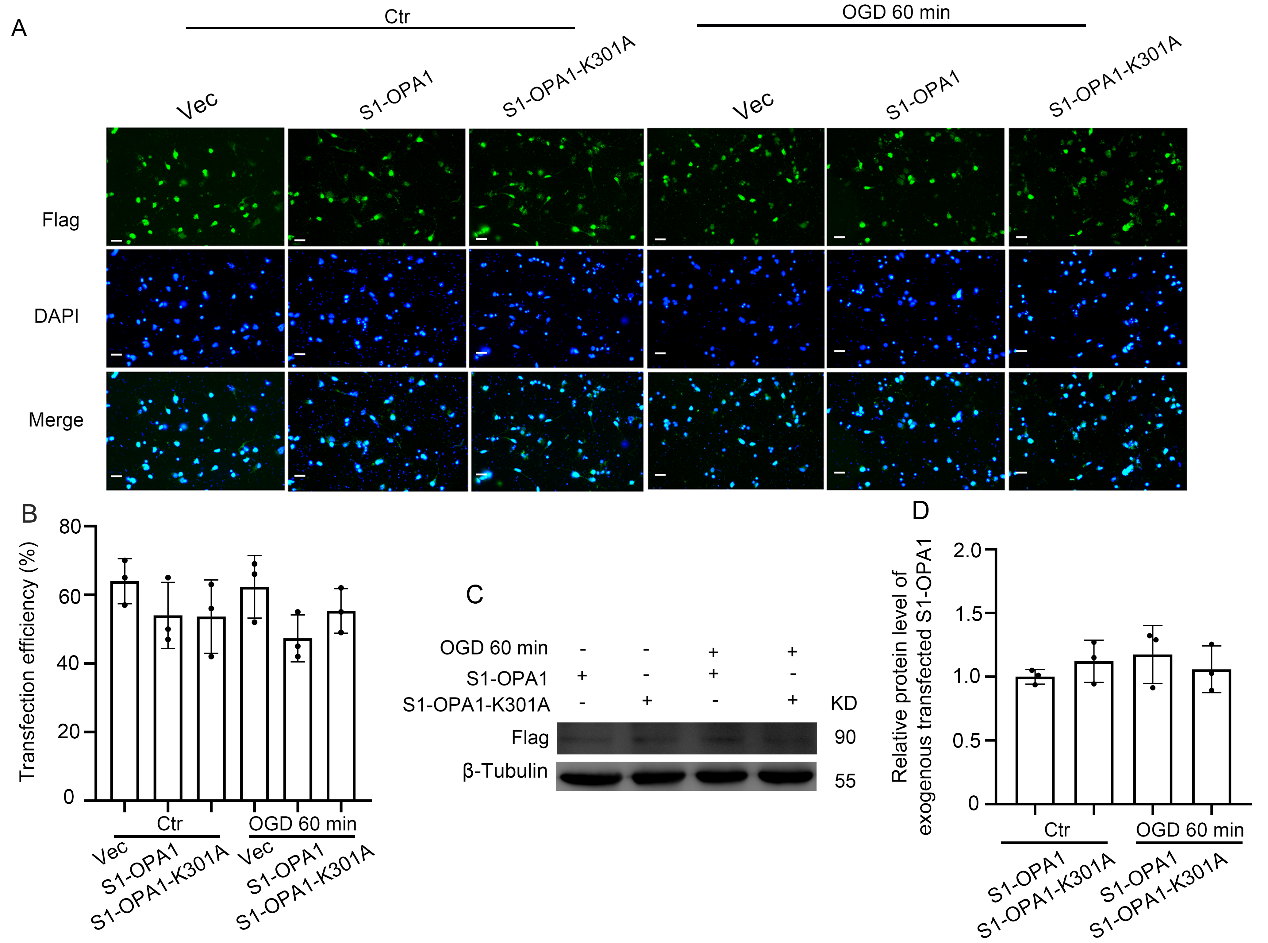
**

**Figure S2. Transfection efficiency of lentivirus in primary cultured neurons.** (A, B) Immunofluorescent analysis of the transfection efficiency 3 days after transfection in cultured neurons under normal condition and OGD for 60 min. (C, D) Western blot analysis and quantification of LV-S1-OPA1-Flag and LV-S1-OPA1-K301A-Flag in cultured neurons under normal condition and OGD for 60 min. Scale bar = 20 μm; n = 3.

**Figure S3**

**
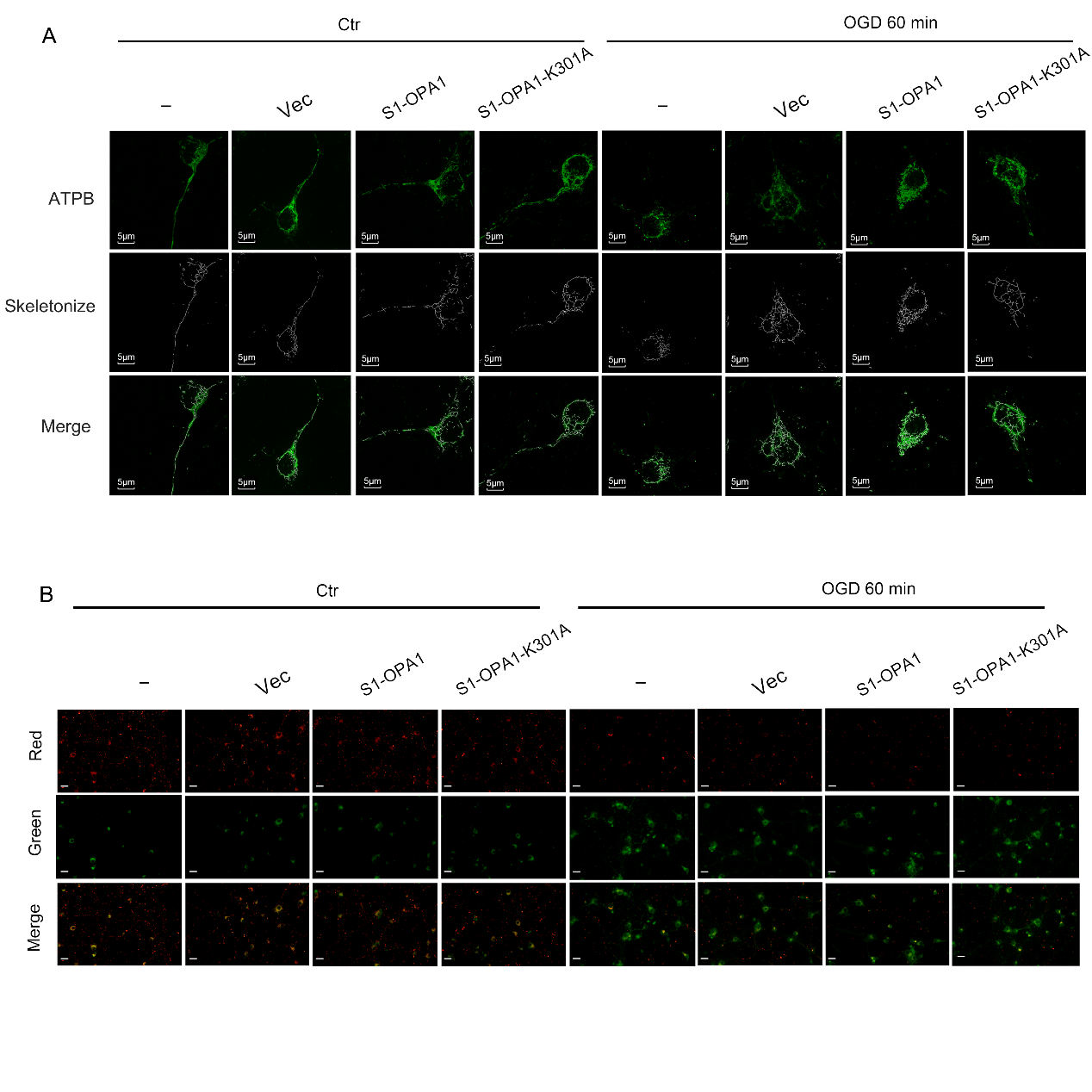
Figure S3. Overexpression of S1-OPA1 exacerbated mitochondrial fragmentation and membrane potential damage after OGD.** (A) Representative confocal images of mitochondrial morphologies in different overexpression groups under normal condition and OGD for 60 min. (B) Representative images of JC-1 fluorescence staining in different overexpression groups under normal condition and OGD for 60 min. Scale bar = 20 μm.

**Figure S4**

**
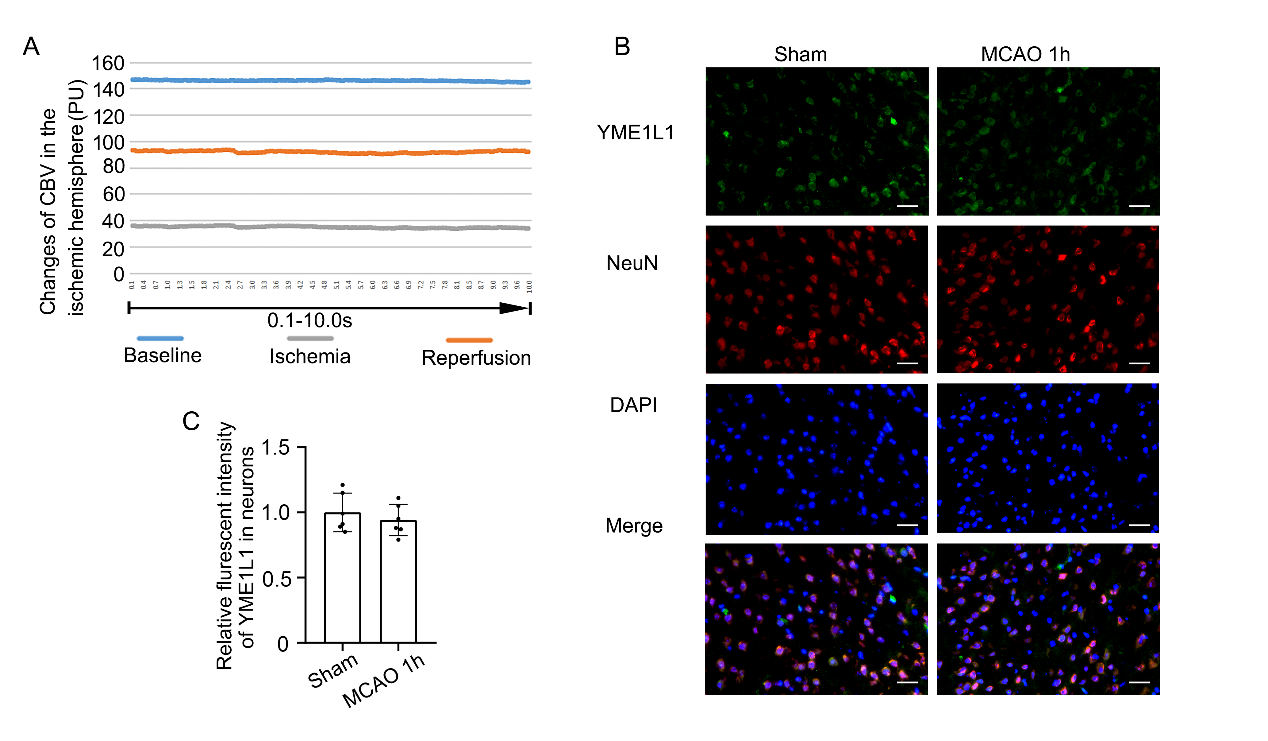
**

**Figure S4. Changes of rCBF during MCAO/R model and YME1L1 in brain tissue surrounding ischemia.** (A) Changes in rCBF in the hemisphere of ischemia and reperfusion. The unit of the recorded value is Perfusion Unit (PU). Blue line represents preoperative baseline, gray line represents ischemia, and the orange line represents reperfusion. (B) Double immunofluorescence analysis was performed with anti-YME1L1 (green) and neuronal marker (NeuN, red) in brain sections. Nuclei were fluorescently labeled with DAPI (blue). (C) The relative fluorescent intensities of YME1L1 in neurons were statistically analyzed.

**Figure S5**

**
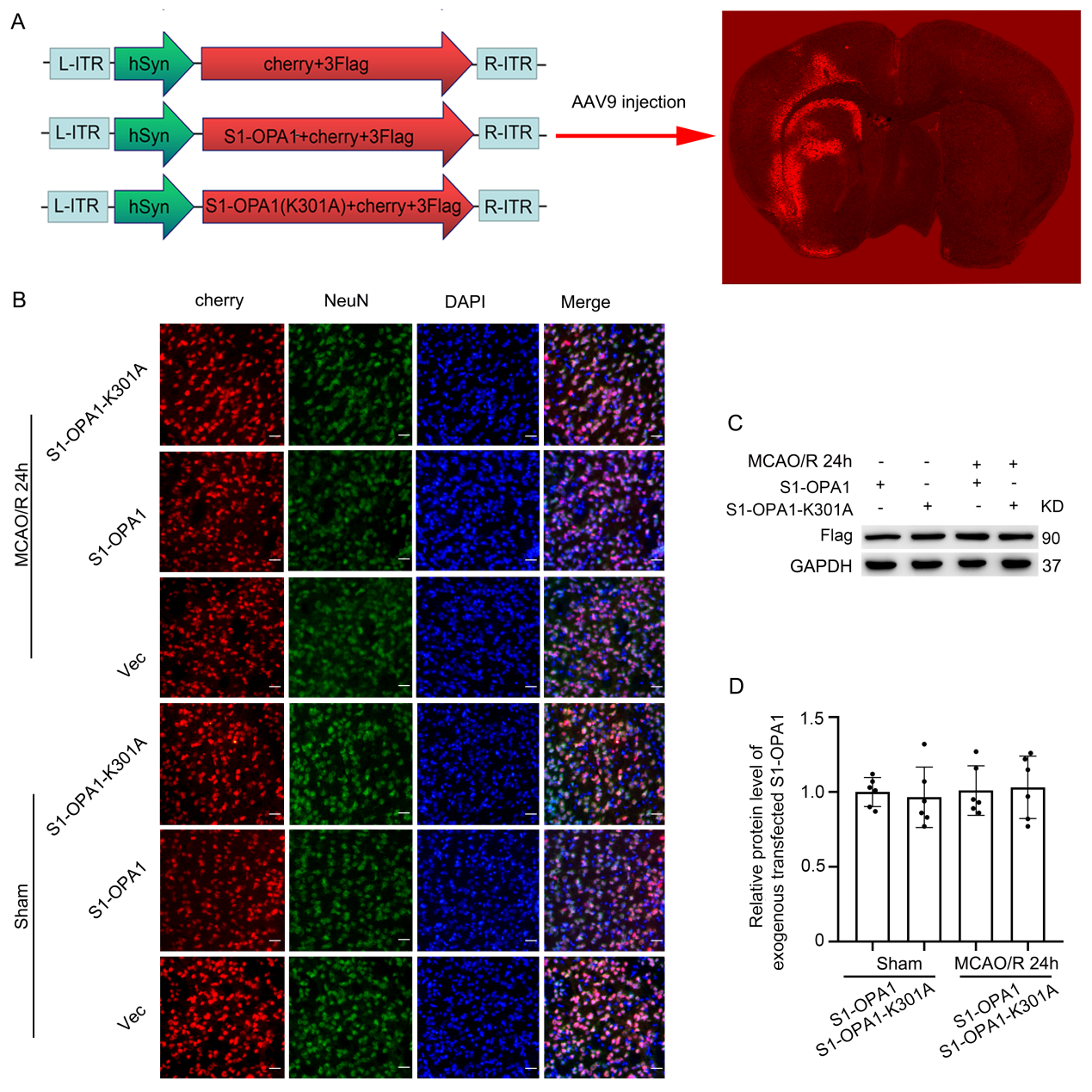
**

**Figure S5. Transfection efficiency of neuron-specific AAVs in mice.** (A) Schematic diagram of the AAV construction. At 21 days after AAV injection, brain section was observed under fluorescent microscope. (B) Immunofluorescence staining was used to label neurons in the transfected regions. Cells transfected with AAVs appeared red (cherry). Neurons were marked in green (NeuN), and nuclei were labeled with blue (DAPI). (C, D) Western blot analysis and quantification of AAV-S1-OPA1-Flag and AAV-S1-OPA1-K301A-Flag in mice. Scale bar = 50 μm; n = 6.

**Figure S6**

**
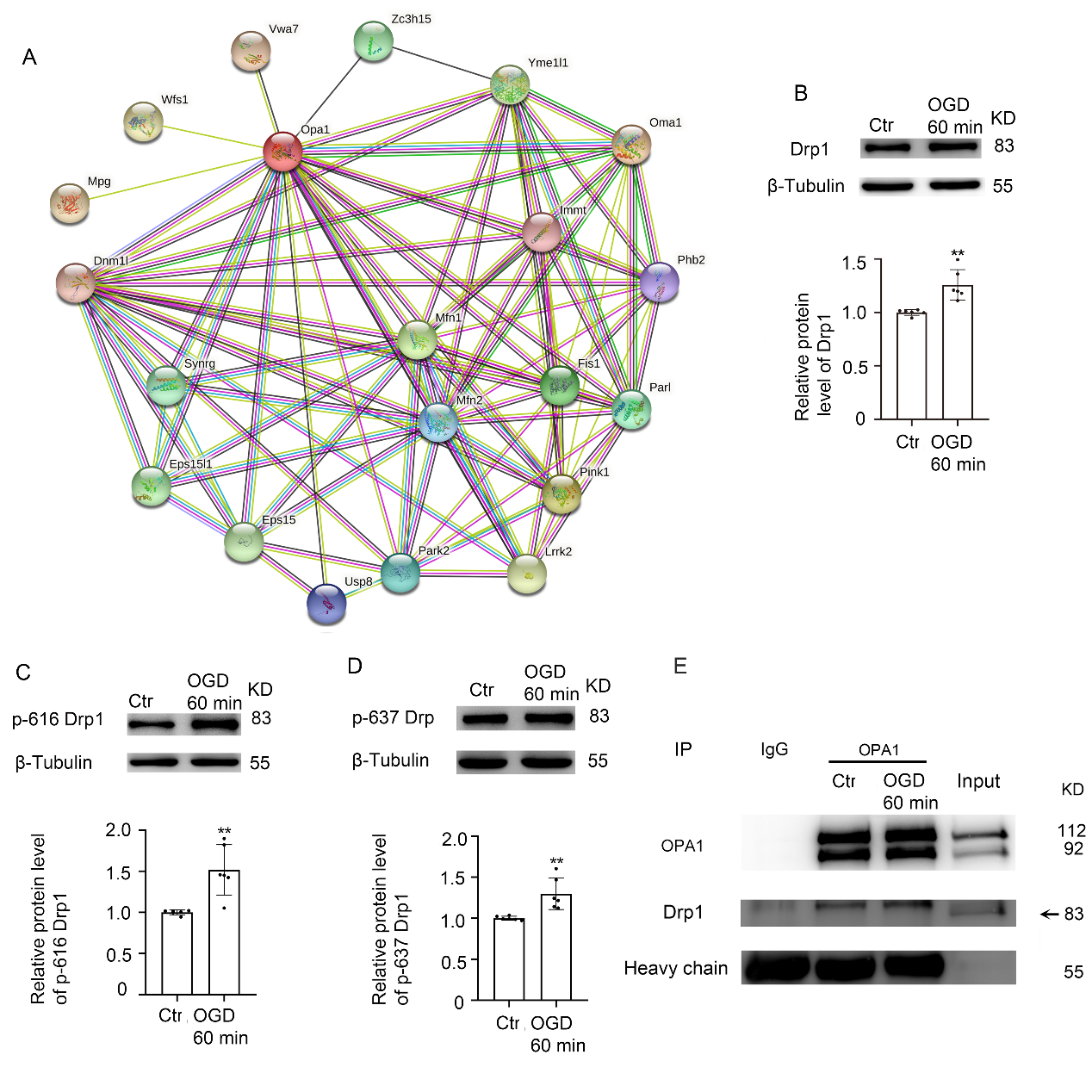
**

**Figure S6. Protein level and phosphorylation level of Drp1 and the interaction with OPA1 after OGD.** (A) Protein network that may interact with OPA1 was identified by the STRING system. (B) Western blot analysis and quantification of Drp1 levels in cultured neurons. (C, D) Western blot analysis and quantification of P-616 Drp1 and P-637 Drp1 levels in cultured neurons. (E) Co-immunoprecipitation (CO-IP) of OPA1 and Drp1 in cultured neurons. ∗∗p < 0.01 vs. control group; n = 6.

**Figure S7**

**
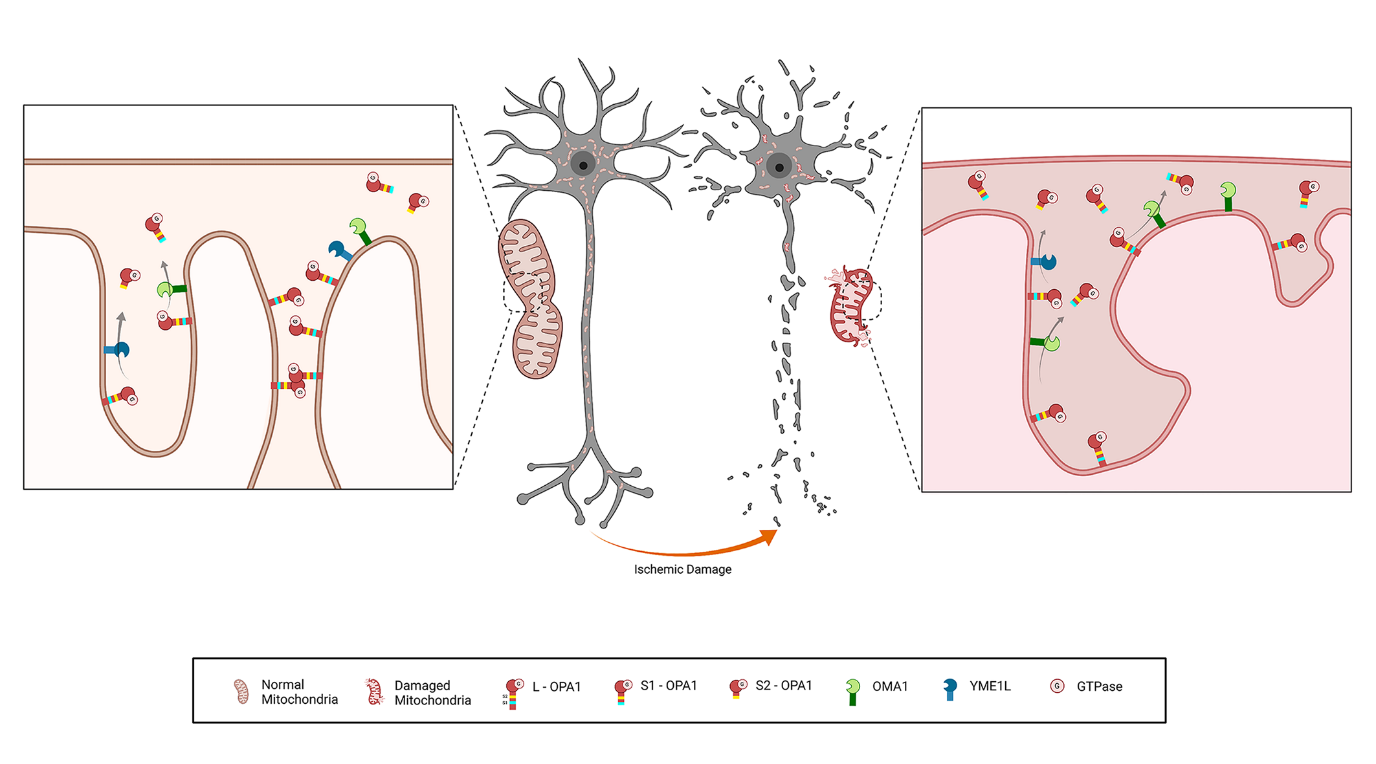
**

**Figure S7. The schema diagram displayed in this study.** Ischemia induced an increase in S1-OPA1 cleaved by OMA1 in neurons and aggravates neuronal mitochondrial fragmentation and functional damage in a GTPase-dependent manner, and participates in ischemia-reperfusion injury.

**Table S1: Specific information about antibodies.**

| Antibody  name | Anti-ATPB antibody | Anti-OPA1 antibody | Anti-OMA1 antibody | Anti-YME1L1 antibody | Anti-Flag antibody | Anti-Caspase-9 antibody | Anti-NeuN antibody | Anti-OMA1 antibody | Anti-Drp1 antibody | Anti-p616-Drp1 antibody | Anti-p637-Drp1 antibody |
| --- | --- | --- | --- | --- | --- | --- | --- | --- | --- | --- | --- |
| Target  antigen | Mouse, Rat, Cow, Human, Monkey | Mouse, Rat, Human | Mouse, Rat, Human | Mouse, Rat, Human | All species | Mouse, Rat | Mouse, Rat, Human | Mouse, Rat, Human | Mouse, Rat, Human | Mouse, Rat, Human | Mouse, Rat, Human |
| Vendor | Abcam | Abcam | Abcam | Proteintech | Cell Signaling Technology | Abcam | Abcam | Affinity | Cell Signaling Technology | Cell Signaling Technology | Cell Signaling Technology |
| Cat number | ab14730 | ab42364 | ab154949 | 11510-1-AP | 8146 | ab184786 | ab104224 | DF12435 | 5391 | 4494 | 6319 |
| Clonality | Monoclonal  antibody | Polyclonal  antibody | Polyclonal  antibody | Polyclonal  antibody | Monoclonal  antibody | Monoclonal  antibody | Monoclonal  antibody | Polyclonal  antibody | Monoclonal | Monoclonal | Monoclonal |
| Host  Organism | Mouse | Rabbit | Rabbit | Rabbit | Mouse | Rabbit | Mouse | Rabbit | Rabbit | Rabbit | Rabbit |
| Comments | WB, ICC/IF, Flow Cyt, IHC-P | WB, IP | WB, IHC-P | WB, IP, IHC, IF, ELISA | WB, IP, IHC, IF | WB, IP | IHC, IF, WB | WB, IHC, IF/ICC | WB, IP | WB, IF | WB |
| Applicate  dilution | IF=1:300 | WB=1:1000  IP=1:200 | WB=1:1000 | WB=1:1000 | WB=1:1000  IF=1:25 | WB=1:1000 | IF=1:300 | IF=1:100 | WB=1:1000 | WB=1:1000 | WB=1:1000 |

**Table S2: Gene regulation of S1-OPA1.**

Gene name: OPA1(mitochondrial dynamin like GTPase); Gene ID: 74143; Species: mouse

S1-OPA1 sequence

| ATGTGGCGAGCAGGTCGGGCGGCCGTGGCCTGTGAAGTCTGCCAATCCTTAGTGAAACACAGTTCTGGAATACAAAGAAACGTACCGCTCCAAAAACTCCATCTGGTTTCACGAAGTATTTATCGTTCACATCATCCTGCCCTCAAGCTTCAAAGACCCCAACTAAGGACACCATTTCAGCAGTTCTCTTCTCTAACTCACCTTTCATTACATAAATTGAAACTTTCTCCAATTAAATATGGCTACCAGCCCCGCAGGAACGCAACAGATCATGGATCTGAAAGTGACAAGCATTACAGGAAGGTGTCAGACAAAGAAAAGATTGACCAACTTCAAGAAGAACTTCTGCATACTCAGTTAAAGTATCAGAGGATCTTGGAGCGCCTGGAAAAGGAGAACAAAGAGCTGCGGAAGCTGGTGCTGCAGAAGGACGACAAAGGCATCCACCACAGGAAGCTCAAGAAATCTTTGATTGATATGTATTCTGAAGTTCTTGATGTTCTTTCTGATTATGATGCCAGTTACAATACACAAGATCACCTACCACGGGTTGTTGTGGTTGGAGATCAGAGTGCTGGGAAAACCAGTGTGCTGGAAATGATTGCTCAGGCCCGGATCTTCCCGAGAGGGTCCGGCGAGATGATGACACGCTCTCCAGTGAAGGTGACTCTCAGTGAAGGCCCTCACCATGTGGCCTTGTTTAAAGATAGCTCTCGGGAATTTGATCTCACCAAGGAGGAAGATCTTGCAGCATTAAGACATGAAATCGAACTCCGAATGAGGAAAAATGTGAAAGAAGGTTGTACTGTTAGCCCCGAGACCATATCTCTAAATGTCAAAGGCCCTGGGCTGCAGAGGATGGTGCTCGTGGACTTGCCTGGTGTCATCAACACCGTGACATCAGGCATGGCTCCCGACACAAAGGAAACTATTTTCAGTATCAGCAAAGCTTACATGCAGAATCCTAACGCCATCATCCTGTGCATCCAAGACGGATCCGTAGATGCTGAGCGCAGTATTGTTACAGACTTGGTCAGTCAAATGGATCCTCATGGAAGAAGAACCATATTTGTTTTGACCAAAGTAGACCTGGCAGAAAAAAATGTAGCCAGTCCAAGCAGGATACAACAGATAATTGAAGGCAAGCTCTTCCCAATGAAAGCTCTGGGTTATTTTGCTGTCGTAACAGGAAAAGGAAACAGCTCTGAAAGTATTGAAGCTATAAGAGAGTATGAAGAGGAATTTTTTCAGAATTCAAAACTGCTAAAGACAAGCATGCTAAAGGCACACCAGGTCACCACGAGAAATCTCAGCCTTGCTGTGTCAGACTGCTTTTGGAAAATGGTTCGAGAGTCAGTTGAACAACAGGCTGATAGTTTTAAAGCCACGCGCTTTAACCTAGAGACGGAATGGAAGAATAACTACCCGCGCCTGCGAGAGCTCGACAGGAATGAACTCTTTGAAAAAGCTAAAAATGAGATCCTCGATGAGGTCATCAGTCTGAGCCAGGTCACGCCAAAGCACTGGGAGGAAATCCTGCAGCAATCCCTGTGGGAACGAGTGTCAACACATGTGATTGAGAACATCTACCTTCCAGCTGCCCAGACCATGAATTCGGGAACATTTAACACCACAGTAGACATCAAGCTTAAACAGTGGACTGACAAGCAGCTTCCTAATAAAGCAGTCGAGGTTGCCTGGGAGACTCTACAAGAGGAATTTTCCCGCTTCATGACAGAACCCAAAGGAAAGGAACACGACGACATATTTGACAAACTTAAGGAGGCTGTGAAGGAGGAGAGTATCAAGCGGCACAAGTGGAACGACTTTGCCGAGGATAGCTTGAGGGTTATTCAGCACAATGCTTTGGAAGACCGGTCCATATCAGATAAGCAACAGTGGGATGCAGCCATTTACTTCATGGAAGAGGCGCTTCAAGGTCGTCTCAAGGATACTGAAAATGCTATTGAAAACATGATTGGGCCAGACTGGAAAAAGAGGTGGATGTACTGGAAGAATCGGACCCAAGAGCAGTGTGTTCACAACGAAACCAAGAACGAGTTGGAGAAGATGCTGAAGGTTAATGATGAGCACCCAGCTTACCTGGCAAGTGATGAGATTACCACAGTCCGGAAGAACCTGGAGTCTCGAGGAGTGGAAGTCGATCCAAGCTTGATTAAGGATACTTGGCATCAAGTTTATAGAAGACATTTCTTAAAAACAGCTCTAAATCATTGTAACCTTTGTCGCAGAGGTTTTTATTACTACCAGAGGCATTTTATAGATTCTGAGCTGGAATGCAATGACGTGGTCCTGTTTTGGCGAATACAGCGCATGCTCGCTATCACTGCCAATACATTAAGGCAGCAGCTTACAAACACTGAAGTTAGGCGACTAGAGAAAAACGTTAAAGAGGTATTAGAAGATTTTGCAGAAGACGGTGAGAAGAAGGTTAAATTGCTCACTGGCAAACGAGTTCAGCTGGCAGAAGATCTCAAGAAAGTTAGAGAAATTCAAGAAAAGCTTGATGCTTTCATTGAAGCTCTTCACCAGGAGAAG |
| --- |

S1-OPA1 (K301A) sequence

| ATGTGGCGAGCAGGTCGGGCGGCCGTGGCCTGTGAAGTCTGCCAATCCTTAGTGAAACACAGTTCTGGAATACAAAGAAACGTACCGCTCCAAAAACTCCATCTGGTTTCACGAAGTATTTATCGTTCACATCATCCTGCCCTCAAGCTTCAAAGACCCCAACTAAGGACACCATTTCAGCAGTTCTCTTCTCTAACTCACCTTTCATTACATAAATTGAAACTTTCTCCAATTAAATATGGCTACCAGCCCCGCAGGAACGCAACAGATCATGGATCTGAAAGTGACAAGCATTACAGGAAGGTGTCAGACAAAGAAAAGATTGACCAACTTCAAGAAGAACTTCTGCATACTCAGTTAAAGTATCAGAGGATCTTGGAGCGCCTGGAAAAGGAGAACAAAGAGCTGCGGAAGCTGGTGCTGCAGAAGGACGACAAAGGCATCCACCACAGGAAGCTCAAGAAATCTTTGATTGATATGTATTCTGAAGTTCTTGATGTTCTTTCTGATTATGATGCCAGTTACAATACACAAGATCACCTACCACGGGTTGTTGTGGTTGGAGATCAGAGTGCTGGGGCCACCAGTGTGCTGGAAATGATTGCTCAGGCCCGGATCTTCCCGAGAGGGTCCGGCGAGATGATGACACGCTCTCCAGTGAAGGTGACTCTCAGTGAAGGCCCTCACCATGTGGCCTTGTTTAAAGATAGCTCTCGGGAATTTGATCTCACCAAGGAGGAAGATCTTGCAGCATTAAGACATGAAATCGAACTCCGAATGAGGAAAAATGTGAAAGAAGGTTGTACTGTTAGCCCCGAGACCATATCTCTAAATGTCAAAGGCCCTGGGCTGCAGAGGATGGTGCTCGTGGACTTGCCTGGTGTCATCAACACCGTGACATCAGGCATGGCTCCCGACACAAAGGAAACTATTTTCAGTATCAGCAAAGCTTACATGCAGAATCCTAACGCCATCATCCTGTGCATCCAAGACGGATCCGTAGATGCTGAGCGCAGTATTGTTACAGACTTGGTCAGTCAAATGGATCCTCATGGAAGAAGAACCATATTTGTTTTGACCAAAGTAGACCTGGCAGAAAAAAATGTAGCCAGTCCAAGCAGGATACAACAGATAATTGAAGGCAAGCTCTTCCCAATGAAAGCTCTGGGTTATTTTGCTGTCGTAACAGGAAAAGGAAACAGCTCTGAAAGTATTGAAGCTATAAGAGAGTATGAAGAGGAATTTTTTCAGAATTCAAAACTGCTAAAGACAAGCATGCTAAAGGCACACCAGGTCACCACGAGAAATCTCAGCCTTGCTGTGTCAGACTGCTTTTGGAAAATGGTTCGAGAGTCAGTTGAACAACAGGCTGATAGTTTTAAAGCCACGCGCTTTAACCTAGAGACGGAATGGAAGAATAACTACCCGCGCCTGCGAGAGCTCGACAGGAATGAACTCTTTGAAAAAGCTAAAAATGAGATCCTCGATGAGGTCATCAGTCTGAGCCAGGTCACGCCAAAGCACTGGGAGGAAATCCTGCAGCAATCCCTGTGGGAACGAGTGTCAACACATGTGATTGAGAACATCTACCTTCCAGCTGCCCAGACCATGAATTCGGGAACATTTAACACCACAGTAGACATCAAGCTTAAACAGTGGACTGACAAGCAGCTTCCTAATAAAGCAGTCGAGGTTGCCTGGGAGACTCTACAAGAGGAATTTTCCCGCTTCATGACAGAACCCAAAGGAAAGGAACACGACGACATATTTGACAAACTTAAGGAGGCTGTGAAGGAGGAGAGTATCAAGCGGCACAAGTGGAACGACTTTGCCGAGGATAGCTTGAGGGTTATTCAGCACAATGCTTTGGAAGACCGGTCCATATCAGATAAGCAACAGTGGGATGCAGCCATTTACTTCATGGAAGAGGCGCTTCAAGGTCGTCTCAAGGATACTGAAAATGCTATTGAAAACATGATTGGGCCAGACTGGAAAAAGAGGTGGATGTACTGGAAGAATCGGACCCAAGAGCAGTGTGTTCACAACGAAACCAAGAACGAGTTGGAGAAGATGCTGAAGGTTAATGATGAGCACCCAGCTTACCTGGCAAGTGATGAGATTACCACAGTCCGGAAGAACCTGGAGTCTCGAGGAGTGGAAGTCGATCCAAGCTTGATTAAGGATACTTGGCATCAAGTTTATAGAAGACATTTCTTAAAAACAGCTCTAAATCATTGTAACCTTTGTCGCAGAGGTTTTTATTACTACCAGAGGCATTTTATAGATTCTGAGCTGGAATGCAATGACGTGGTCCTGTTTTGGCGAATACAGCGCATGCTCGCTATCACTGCCAATACATTAAGGCAGCAGCTTACAAACACTGAAGTTAGGCGACTAGAGAAAAACGTTAAAGAGGTATTAGAAGATTTTGCAGAAGACGGTGAGAAGAAGGTTAAATTGCTCACTGGCAAACGAGTTCAGCTGGCAGAAGATCTCAAGAAAGTTAGAGAAATTCAAGAAAAGCTTGATGCTTTCATTGAAGCTCTTCACCAGGAGAAG |
| --- |

Vector information in lentiviruses and adeno-associated viruses:

Vector name in lentiviruses: GV341; Element sequence: Ubi-MCS-3FLAG-SV40-puromycin; Clonal site: AgeI/NheI

Vector atlas:

**
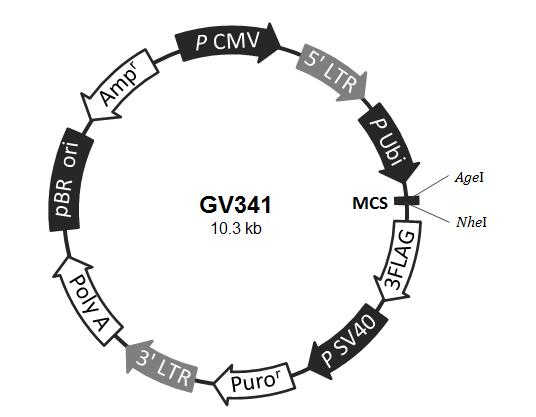
**

Vector name in adeno-associated viruses: GV475; Element sequence: hSyn promoter-MCS-cherry-3FLAG-SV40 PolyA; Clonal site: EcoRI/BamHI

Vector atlas:


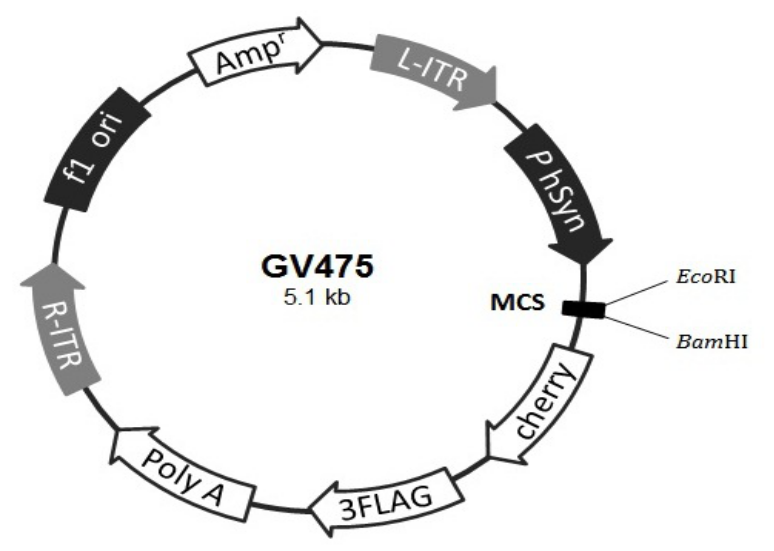


**Table S3: Detailed statistics.**

| Figures | Description | Testing time | In vivo or in vitro | Test used | Detail value | One- or two- tailed P value? |
| --- | --- | --- | --- | --- | --- | --- |
| Figure 1B | Neurons with fragmented mitochondria (%) | OGD 30min -60min | In vitro | One-way ANOVA | Ctr vs. OGD 30 min: Mean Diff = -21.33, 95% CI=-36.34 to -6.324, P=0.0117,  Ctr vs. OGD 60 min: Mean Diff =-53.67, 95% CI=-68.68 to -38.66, P=0.0001 | Two-tailed |
| Figure 1C | Relative protein level of OPA1 | OGD  60 min | In vitro | Multiple t test | Ctr vs. OGD 60 min:  T-OPA1: Mean Diff =0.1387, P=0.080123, L-OPA1: Mean Diff =0.1322, P=0.12224, S-OPA1: Mean Diff =0.2726, P=0.026022 | Two-tailed |
| Figure 1D | Relative protein level of OMA1 | OGD  60 min | In vitro | Unpaired t test | Ctr vs. OGD 60 min: Mean Diff=0.3926, 95% CI=0.08970 to 0.6954, P=0.0001 | Two-tailed |
| Figure 1E | Relative protein level of YME1L1 | OGD  60 min | In vitro | Unpaired t test | Ctr vs. OGD 60 min: Mean Diff =-0.1133, 95% CI=-0.2356 to 0.008934, P=0.0658 | Two-tailed |
| Figure 2B | Neurons with fragmented mitochondria (%) | OGD  60 min | In vitro | One-way ANOVA | Ctr vs. OGD 60 min: Mean Diff = -49.33, 95% CI=-62.05 to -36.61, p<0.0001,  OGD 60 min Vec vs. OGD 60 min S1-OPA1: Mean Diff =-18.33, 95% CI=-31.05 to -5.613, P=0.0026,  OGD 60 min S1-OPA1 vs. OGD 60 min S1-OPA1-K301A: Mean Diff =15.33, 95% CI=2.613 to 28.05, P=0.0128 | Two-tailed |
| Figure 2D | JC-1 fluorescence intensity ratio (Red/Green) | OGD  60 min | In vitro | One-way ANOVA | Ctr vs. OGD 60 min: Mean Diff = 0.8933, 95% CI=0.7040 to 1.083, p<0.0001,  OGD 60 min +Vec vs. OGD 60 min S1-OPA1: Mean Diff =0.2167, 95% CI=0.02730 to 0.4060, P=0.0193,  OGD 60 min S1-OPA1 vs. OGD 60 min S1-OPA1-K301A: Mean Diff =-0.2100, 95% CI=-0.3994 to -0.02063, P=0.0244 | Two-tailed |
| Figure 3B | Percentage of mitochondrial with dense and regular cristate (%) | OGD/R 24h | In vitro | One-way ANOVA | Ctr vs. OGD/R 24h: Mean Diff = 54, 95% CI=40.42 to 67.58, p<0.0001,  OGD/R 24h Vec vs. OGD/R 24h S1-OPA1: Mean Diff =14, 95% CI=1.751to 26.25, P=0.0248,  OGD/R 24h S1-OPA1 vs. OGD/R 24h S1-OPA1-K301A: Mean Diff =-12.67, 95% CI=-24.92to-0.4181, P=0.0422 | Two-tailed |
| Figure 3C | Mitochondrial length (um) | OGD/R 24h | In vitro | One-way ANOVA | Ctr vs. OGD/R 24h: Mean Diff = 0.45, 95%CI=0.1608 to 0.7392, p=0.0012.OGD/R 24h Vec vs. OGD/R 24h S1-OPA1: Mean Diff =0.3633, 95%CI=0.7413to 0.6525, P=0.0091,  OGD/R 24h S1-OPA1 vs. OGD/R 24h S1-OPA1-K301A: Mean Diff =-0.3467, 95% CI=-0.6359to-0.0574, P=0.0134 | Two-tailed |
| Figure 4B | Relative mitochondrial superoxide | OGD/R 24h | In vitro | One-way ANOVA | Ctr vs. OGD/R 24h: Mean Diff = -3.167, 95% CI=-4.938 to -1.396, p=0.0003  OGD/R 24h Vec vs. OGD/R 24h S1-OPA1: Mean Diff =-2.400, 95% CI=-4.171 to -0.6290, P=0.0046  OGD/R 24h S1-OPA1 vs. OGD/R 24h S1-OPA1-K301A: Mean Diff =2.133, 95% CI=0.3623 to 3.904, P=0.0128 | Two-tailed |
| Figure 4C | ATP concentration (umol/g) | OGD/R 24h | In vitro | One-way ANOVA | Ctr vs. OGD/R 24h: Mean Diff = 4.507, 95% CI=3.189 to 5.825, p<0.0001  OGD/R 24h Vec vs. OGD/R 24h S1-OPA1: Mean Diff =1.503, 95% CI=0.1847 to 2.821, P=0.0198,  OGD/R 24h S1-OPA1 vs. OGD/R 24h S1-OPA1-K301A: Mean Diff =-1.671, 95% CI=-2.989 to -0.3525, P=0.0084 | Two-tailed |
| Figure 4E | Relative cleaved caspase-9/procaspase-9 | OGD/R 24h | In vitro | One-way ANOVA | Ctr vs. OGD/R 24h: Mean Diff =-0.7284, 95% CI=-1.157 to -0.2994, p=0.0005,  OGD/R 24h Vec vs. OGD/R 24h S1-OPA1: Mean Diff =-1.225, 95% CI=-1.654 to -0.7964, P<0.0001  OGD/R 24h S1-OPA1 vs. OGD/R 24h S1-OPA1-K301A: Mean Diff =0.8268, 95% CI=0.3978 to 1.256, P=0.0001 | Two-tailed |
| Figure 5B | Apoptotic neurons (%) | OGD/R 24h | In vitro | One-way ANOVA | Ctr vs. OGD/R 24h: Mean Diff = -20.67, 95% CI=-31.00 to -10.33, p<0.0001  OGD/R 24h Vec vs. OGD/R 24h S1-OPA1: Mean Diff =-16.00, 95% CI=-26.34 to -5.662, P=0.0013  OGD/R 24h S1-OPA1 vs. OGD/R 24h S1-OPA1-K301A: Mean Diff =12.00, 95% CI=1.662 to 22.34, P=0.0172 | Two-tailed |
| Figure 5D | Relative ratio of live neurons (%) | OGD/R 24h | In vitro | One-way ANOVA | Ctr vs. OGD/R 24h: Mean Diff = 40.00, 95% CI=23.31 to 56.69, p<0.0001  OGD/R 24h Vec vs. OGD/R 24h S1-OPA1: Mean Diff =19.33, 95% CI=2.639 to 36.03, P=0.0176,  OGD/R 24h S1-OPA1 vs. OGD/R 24h S1-OPA1-K301A: Mean Diff =-19.00, 95% CI=-35.60 to -2.306, P=0.0201 | Two-tailed |
| Figure 6C | Relative protein level of OPA1 | MCAO 1h | In vivo | Multiple t test | Sham vs. MCAO 1h:  T-OPA1: Mean Diff =0.2017, P=0.1688, L-OPA1: Mean Diff =0.1483, P=0.4083, S-OPA1: Mean Diff =0.3083, P=0.0164 | Two-tailed |
| Figure 6E | Relative fluorescent intensity level of OMA1 in neurons | MCAO 1h | In vivo | Unpaired t test | Sham vs. MCAO 1h:  Mean Diff=0.3983, 95% CI=0.1829 to 0.6138, P=0.0021 | Two-tailed |
| Figure 6G | Percentage of mitochondrial with dense and regular cristate (%) | MCAO/R 24h | In vivo | One-way ANOVA | Sham vs. MCAO/R 24h: Mean Diff = 54, 67% CI=42.90 to 66.44.58, p<0.0001,  MCAO/R 24h Vec vs. MCAO/R 24h S1-OPA1: Mean Diff =16, 95% CI=4.231to 21.77, P=0.0045,  MCAO/R 24h S1-OPA1 vs. OGD/R 24h S1-OPA1-K301A: Mean Diff =-12.33, 95% CI=-24.10to-0.5643, P=0.0366 | Two-tailed |
| Figure 6H | Mitochondrial length (um) | MCAO/R 24h | In vivo | One-way ANOVA | Sham vs. MCAO/R 24h: Mean Diff = 0.6973，95% CI=0.5015 to 0.8932, p<0.0001, MCAO/R 24h Vec vs. MCAO/R 24h S1-OPA1: Mean Diff =0.3958, 95%CI=0.2to 0.5916, p<0.0001,  MCAO/R 24h S1-OPA1 vs. MCAO/R 24h S1-OPA1-K301A: Mean Diff =-0.2141, 95% CI=-0.41to-0.01831, P=0.027 | Two-tailed |
| Figure 7B | FJC - positive neurons (mm^2^) | MCAO/R 24h | In vivo | One-way ANOVA | Sham vs. MCAO/R 24h: Mean Diff = -179.5，95% CI= -291.3 to -139.7, p<0.0001, MCAO/R 24h Vec vs. MCAO/R 24h S1-OPA1: Mean Diff =-79.0, 95%CI=-118.8 to -39.21, p<0.0001,  MCAO/R 24h S1-OPA1 vs. MCAO/R 24h S1-OPA1-K301A: Mean Diff =89.67, 95% CI=49.88 to 129.5, p<0.0001 | Two-tailed |
| Figure 7C | Brain water content (%) | MCAO/R 24h | In vivo | Two-way ANOVA | Sham Vec vs. MCAO/R 24h Vec: Mean Diff = -2.850，95% CI= -3.561 to -2.139, p<0.0001, MCAO/R 24h Vec vs. MCAO/R 24h S1-OPA1: Mean Diff =-1.450, 95%CI=-2.161 to -0.7393, p<0.0001,  MCAO/R 24h S1-OPA1 vs. MCAO/R 24h S1-OPA1-K301A: Mean Diff =1.300, 95% CI=0.5893 to 2.011, p<0.0001 | Two-tailed |
| Figure 7E | Infarct volume (%) | MCAO/R 24h | In vivo | One-way ANOVA | MCAO/R 24h Vec vs. MCAO/R 24h S1-OPA1: Mean Diff =-6.433, 95%CI=-10.46 to -2.408, p=0.0012,  MCAO/R 24h S1-OPA1 vs. MCAO/R 24h S1-OPA1-K301A: Mean Diff =4.717, 95% CI=0.6908 to 8.742, p=0.0181 | Two-tailed |
| Figure 8A | Latency to fall (s) | MCAO/R 1d-28d | In vivo | Two-way ANOVA | Sham vs. MCAO/R 24h: Mean Diff = 53.57，95% CI=45.52 to 61.62, p<0.0001, MCAO/R Vec vs. MCAO/R S1-OPA1: Mean Diff =11.92, 95%CI=3.868 to 19.97, p<0.0001,  MCAO/R S1-OPA1 vs. MCAO/R S1-OPA1-K301A: Mean Diff =-13.81, 95% CI=-21.86to-5.764, p<0.0001 | Two-tailed |
| Figure 8B | Foot fault rate (%) | MCAO/R 1d-28d | In vivo | Two-way ANOVA | Sham vs. MCAO/R 24h: Mean Diff =-9.275，95% CI=-9.609 to-8.941, p<0.0001, MCAO/R Vec vs. MCAO/R S1-OPA1: Mean Diff =-3.299, 95%CI=-3.750 to -2.848, p<0.0001,  MCAO/R S1-OPA1 vs. MCAO/R S1-OPA1-K301A: Mean Diff =3.407, 95% CI=2.957 to 3.858, p<0.0001 | Two-tailed |
| Figure 8C | Time to touch (s) | MCAO/R 1d-28d | In vivo | Two-way ANOVA | Sham vs. MCAO/R 24h: Mean Diff = -11.29，95% CI=-12.87 to-9.711, p<0.0001, MCAO/R Vec vs. MCAO/R S1-OPA1: Mean Diff =-13.20, 95%CI=-14.78 to -11.62, p<0.0001,  MCAO/R S1-OPA1 vs. MCAO/R S1-OPA1-K301A: Mean Diff =13.26, 95% CI=11.68 to 14.84, p<0.0001 | Two-tailed |
| Figure 8D | Time to remove (s) | MCAO/R 1d-28d | In vivo | Two-way ANOVA | Sham vs. MCAO/R 24h: Mean Diff = -29.15，95% CI=-31.40to-26.89, p<0.0001, MCAO/R Vec vs. MCAO/R S1-OPA1: Mean Diff =-23.15, 95%CI=-25.4 to -20.89, p<0.0001,  MCAO/R S1-OPA1 vs. MCAO/R S1-OPA1-K301A: Mean Diff =22.66, 95% CI=20.40 to 24.92, p<0.0001 | Two-tailed |
| Figure S2B | Transfection efficiency (%) | OGD  60 min | In vitro | One-way ANOVA | P summary=0.2296 | Two-tailed |
| Figure S2D | Relative protein level of exogenous transfected S1-OPA1 | OGD  60 min | In vitro | One-way ANOVA | P summary=0.6343 | Two-tailed |
| Figure S4C | Relative fluorescent intensity level of YME1L1 in neurons | MCAO 1h | In vivo | Unpaired t test | Sham vs. MCAO 1h:  Mean Diff=-0.05833, 95% CI=-0.2316 to 0.1150, P=0.4705 | Two-tailed |
| Figure S5D | Relative protein level of exogenous transfected S1-OPA1 | MCAO/R 24h | In vivo | One-way ANOVA | P summary=0.9269 | Two-tailed |
| Figure S6B | Relative protein level of Drp1 | OGD 60 min | In vitro | Unpaired t test | Ctr vs. OGD 60 min: Mean Diff =0.2586, 95% CI=0.1275 to 0.3897, P=0.0013 | Two-tailed |
| Figure S6C | Relative protein level of 616-Drp1 | OGD 60 min | In vitro | Unpaired t test | Ctr vs. OGD 60 min: Mean Diff =0.5171, 95% CI=0.2369 to 0.7972, P=0.0021 | Two-tailed |
| Figure S6D | Relative protein level of 637-Drp1 | OGD 60 min | In vitro | Unpaired t test | Ctr vs. OGD 60 min: Mean Diff =0.2996, 95% CI=0.1224 to 0.4767, P=0.0037 | Two-tailed |
