# Supplementary material for: Ischemia-induced cleavage of OPA1 at S1 site aggravates mitochondrial fragmentation and reperfusion injury in neurons
Source: Cell Death Dis. 2022 Apr 8;13(4):321. doi: 10.1038/s41419-022-04782-0 (PMC8993832; doi:10.1038/s41419-022-04782-0)

**Supplementary materials 2**

Full length uncropped original western blots:

Figure 1 C:


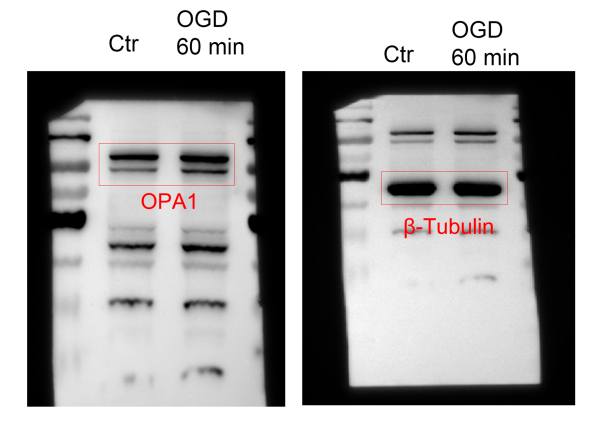


Figure 1 D:


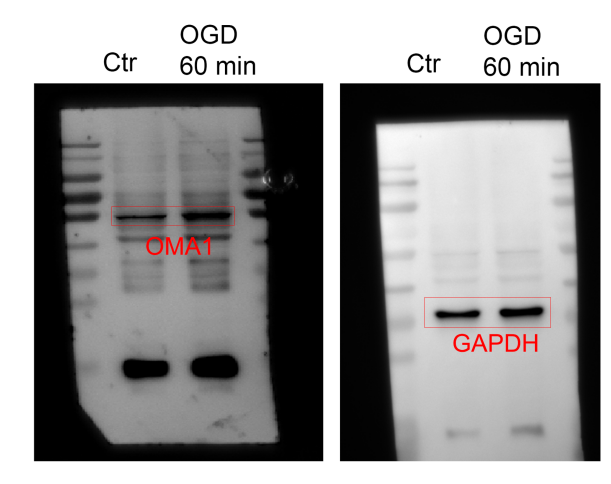


Figure 1 E:


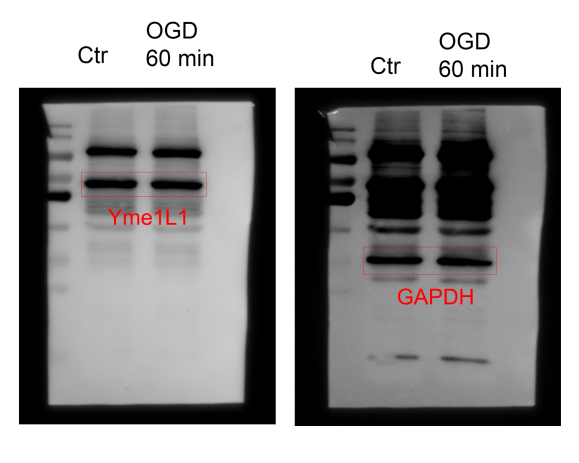


Figure 4 D:


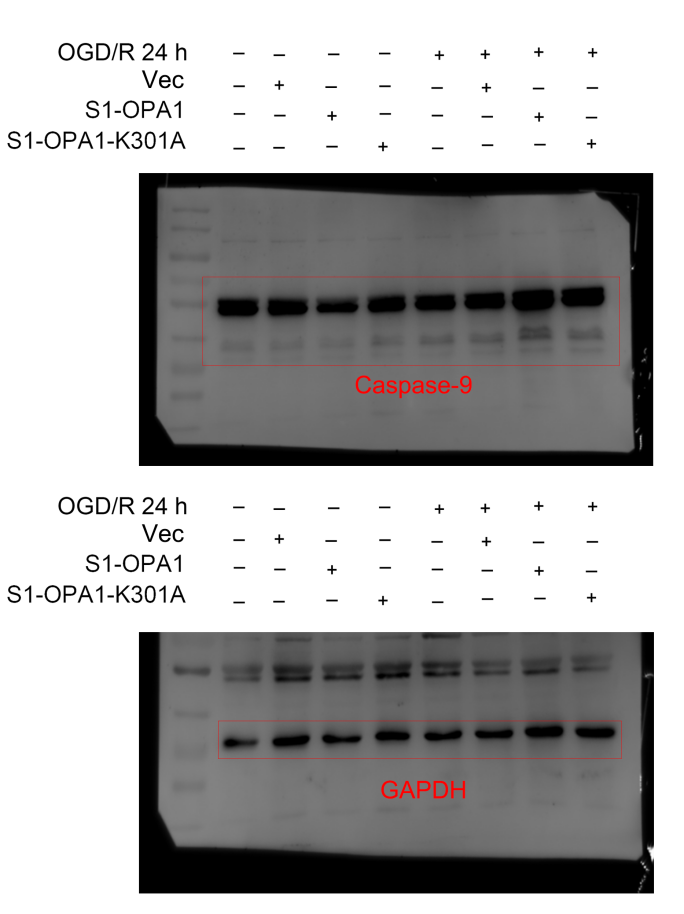


Figure 6 C:


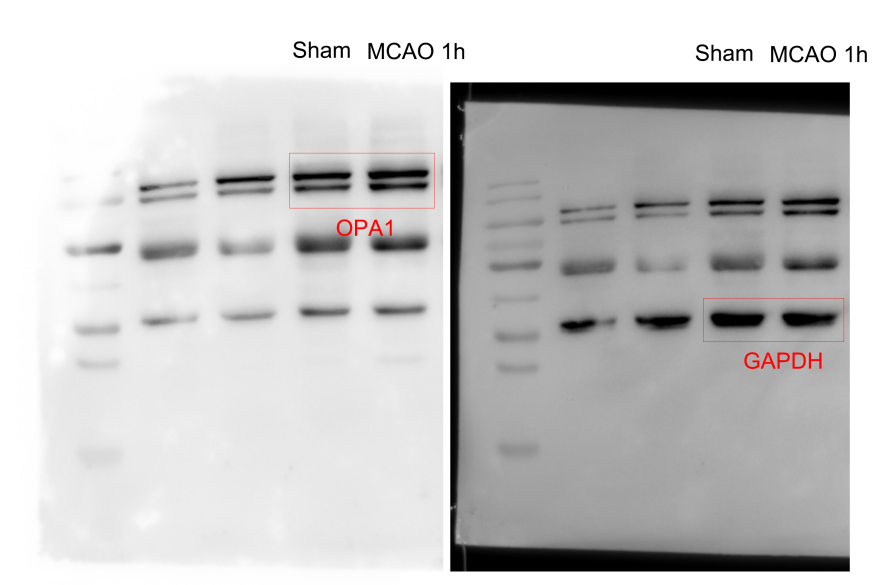


Figure S2 C：


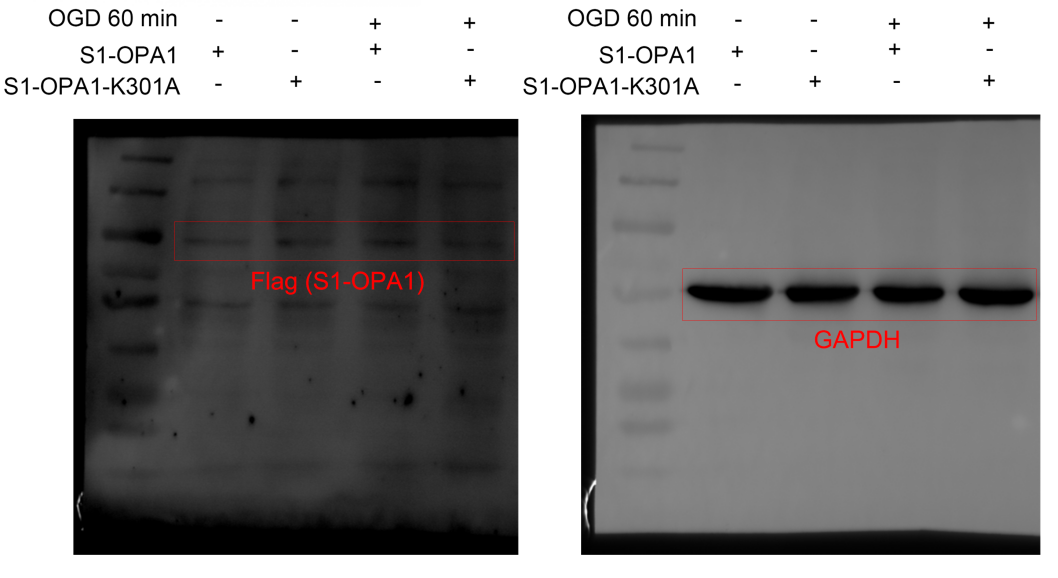


Figure S5 C：


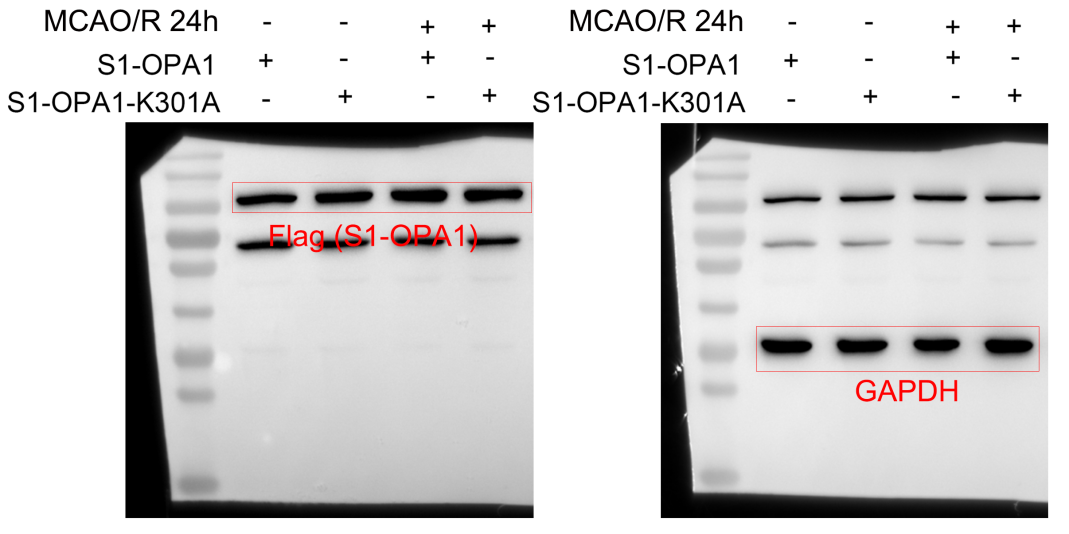


Figure S6 B：


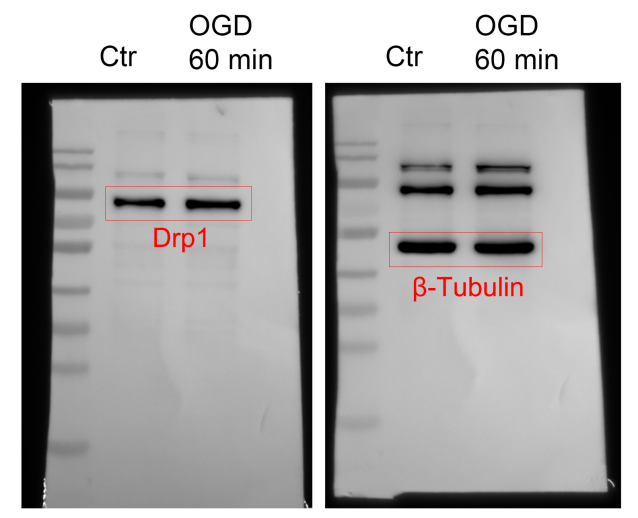


Figure S6 C：


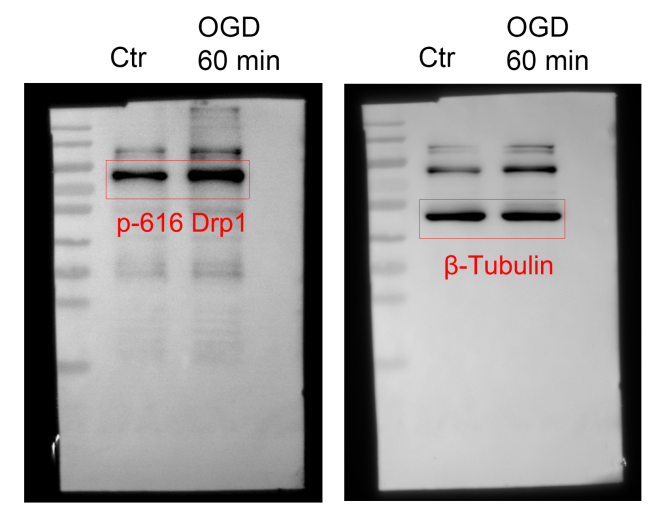


Figure S6 D：


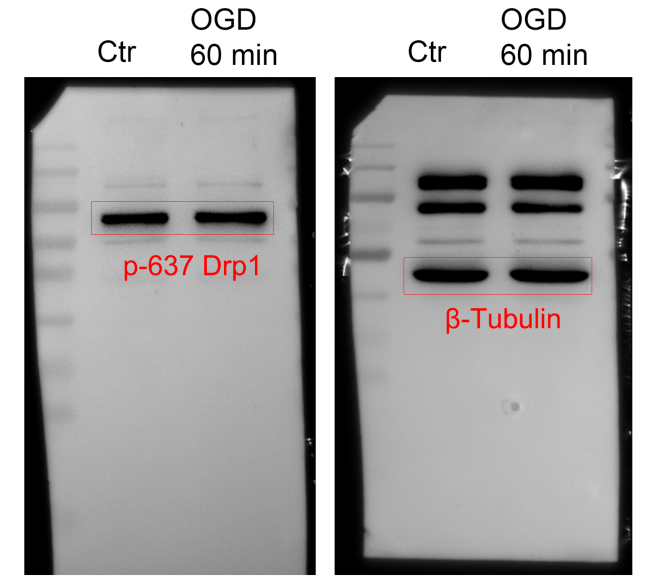


Figure S6 E：


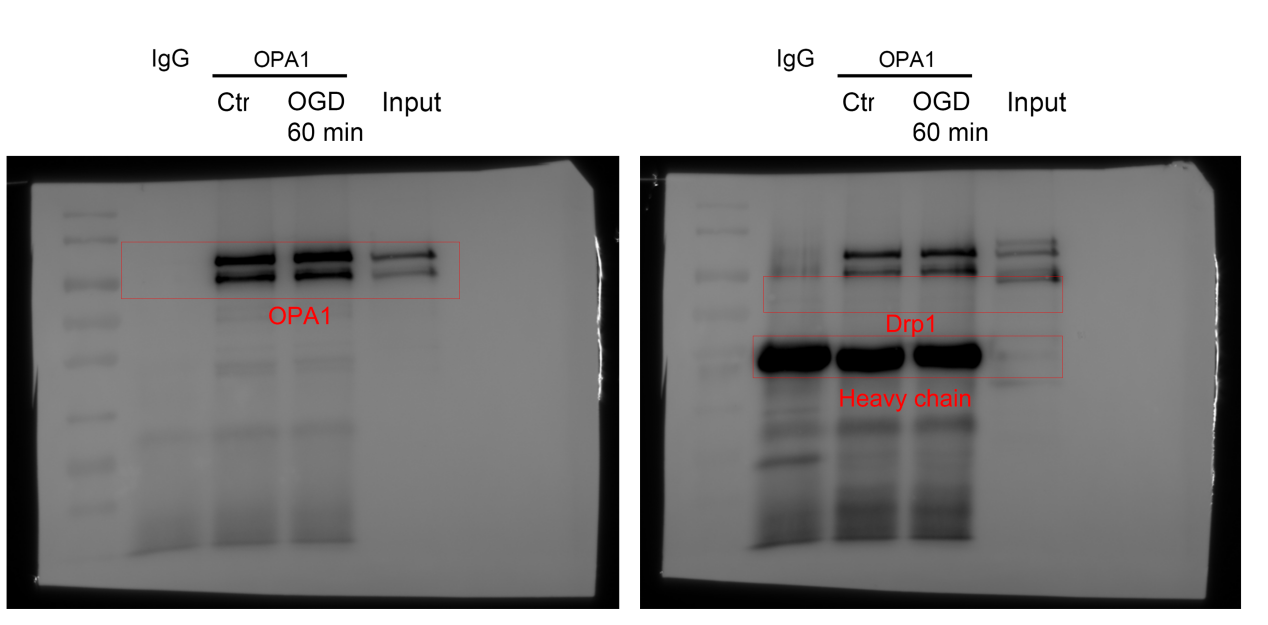

Supplement: Supplementary file 2 — Supplementary materials 2 [file 41419_2022_4782_MOESM2_ESM.docx]
